# Shape-Memory Metallopolymer Networks Based on a Triazole–Pyridine Ligand

**DOI:** 10.3390/polym11111889

**Published:** 2019-11-15

**Authors:** Josefine Meurer, Julian Hniopek, Stefan Zechel, Marcel Enke, Jürgen Vitz, Michael Schmitt, Jürgen Popp, Martin D. Hager, Ulrich S. Schubert

**Affiliations:** 1 Laboratory of Organic and Macromolecular Chemistry (IOMC), Friedrich Schiller University Jena, Humboldstr. 10, 07743 Jena, Germany; josefine.meurer@uni-jena.de (J.M.); stefan.zechel@uni-jena.de (S.Z.); marcel.enke@uni-jena.de (M.E.); j.vitz@uni-jena.de (J.V.); martin.hager@uni-jena.de (M.D.H.); 2 Jena Center of Soft Matter (JCSM), Friedrich Schiller University Jena, Philosophenweg 7, 07743 Jena, Germany; 3 Institute of Physical Chemistry (IPC), Friedrich Schiller University Jena, Helmholzweg 4, 07743 Jena, Germany; julian.hniopek@uni-jena.de (J.H.); m.schmitt@uni-jena.de (M.S.); juergen.popp@uni-jena.de (J.P.); 4 Abbe Center of Photonics, Friedrich Schiller University Jena, Albert-Einstein-Straße 6, 07745 Jena, Germany; 5 Leibniz Institute of Photonic Technology, e. V. Jena, Albert-Einstein-Str. 9, 07745 Jena, Germany

**Keywords:** shape-memory polymer, metallopolymer, triazole-pyridine-metal complex, stimuli responsive polymer

## Abstract

Shape memory polymers represent an interesting class of stimuli-responsive polymers. With their ability to memorize and recover their original shape, they could be useful in almost every area of our daily life. We herein present the synthesis of shape-memory metallopolymers in which the switching unit is designed by using bis(pyridine–triazole) metal complexes. The polymer networks were synthesized via free radical polymerization of methyl-, ethyl- or butyl-methacrylate, tri(ethylene glycol) dimethacrylate and a methacrylate moiety of the triazole–pyridine ligand. By the addition of zinc(II) or cobalt(II) acetate it was possible to achieve metallopolymer networks featuring shape-memory abilities. The successful formation of the metal-ligand complex was proven by Fourier transform infrared (FT-IR) spectroscopy and by ^1^H NMR spectroscopy. Furthermore, the shape-recovery behavior was studied in detailed fashion and even triple-shape memory behavior could be revealed.

## 1. Introduction

Smart materials, in particular smart polymers, gain more and more importance nowadays. These materials are able to change their functions/properties for a predetermined propose after they sensed their own state and/or the environment [1,2,3]. A large variety of polymers is currently known to feature such stimuli-responsive and therefore also smart behavior [4]. Such polymers change their properties during the application of an external trigger [5,6]. Possible triggers are, for example, temperature [7,8,9], pH-value [10,11], light [12,13], electric field [14] and many more [15]. The stimuli-responsive behavior is a precondition for a wide range of different applications such as self-healing behavior [16] or sensor functions and a variety of different properties can be switched, e.g., optical properties [17].

One very important class of smart polymers are shape-memory polymers [18]. These materials have the ability to memorize their original shape (i.e., permanent shape). They can be deformed under certain conditions resulting in one or more new form(s) (i.e., temporary shape(s)), which can be fixed. An external trigger leads to the recovery of the memorized original shape [19,20,21,22,23].

The shape-memory effect was firstly found for shape-memory alloys. In 1963, this ability was obtained for a Ti–Ni alloy [24]. However, polymers were investigated in a more detailed fashion and many different possibilities for shape-memory polymers could be revealed such as light-triggered shape recovery [25].

Shape-memory polymers represent promising materials for numerous potential applications. For instance, they can be utilized in commercial materials, e.g., in textiles or plastic screws [26], for self-healing materials utilizing the so-called shape-memory assisted self-healing (SMASH), which promotes the healing process [27,28], for biomedical applications, e.g., for minimal invasive surgery [29], and in particular in the area of aerospace (e.g., self-deploying antennas) where size are always crucial parameters [30]. For that reason, the latest research on this topic is very versatile. For example, Wang et al. presented a pre-strained 2D shape-memory polymer system, which could be switched to a freestanding 3D architecture [31]. Other groups focused their work on biocompatible SMP foams as new vascular materials for drug-release [32]. Also, new water-responsible polymers were investigated for biomedical applications [33].

The ability for shape-recovery is not particularly based on one specific property of the polymer. It results from the structure as well as the morphology of the whole polymeric system [20]. Thus, shape-memory polymers need at least two different structural units: a stable network, which is responsible for the stability of the whole polymer and determines the permanent shape and a reversible phase enabling the shape-switching. The deformation of the permanent phase is the reason for the shape recovery and, thus, the driving force for the shape-memory behavior. This stable part of the polymer can be generated in three different ways: It is possible to utilize either i) chemical (covalent) [34] or ii) physical (supramolecular) [35] crosslinking; however, iii) interpenetrating networks can also act as the stable network [36]. The second subunit, which is required for shape-memory polymers, must be addressable. This means the second phase is reversible and can be influenced by an external stimulus. This switchable moiety is responsible for the fixation of the temporary shape of the polymer as well as for the possibility to switch between the different shapes [37]. In general, there are many possibilities to design such phase. Possible options include the switching via crystallization/melting [34,38,39,40], transition between different liquid crystalline phases [41], glass transition [42,43], reversible covalent bonds [25,44] and supramolecular interactions [45,46,47]. One example for supramolecular bonds in shape-memory polymers are metal ligand interactions, e.g., utilizing 4-oxy-2,6-bis(*N*-methylbenzimidazolyl)pyridine and an europium salt [46], triphenylphosphine units, which were crosslinked by the addition of rhodium salts [48] or a terpyridine ligand which formed complexes with nickel and iron salts [49]. The latter one represents a rarely studied system even though it features a lot of potential in the context of shape-memory materials. The metal–ligand interactions can be addressed in polymers using a variety of different stimuli and can be fine-tuned according to the strength of the supramolecular unit [50]. Therefore, the different phases of the polymeric systems can be adjusted to the function. However, metallopolymers were rarely investigated in respect to a shape-recovery behavior and general design principles are currently still missing.

**Figure 1:**
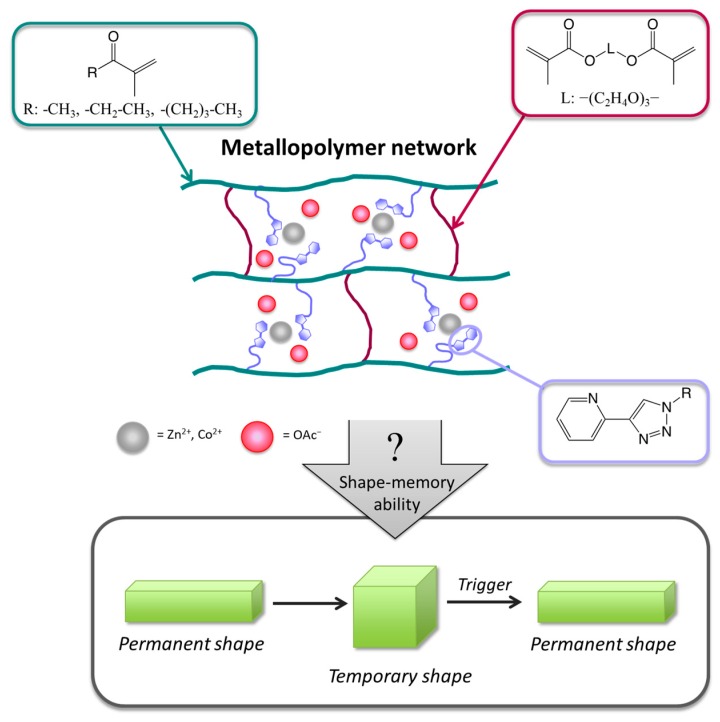
Schematic representation of the concept. Synthesis of metallopolymer networks, starting from different methacrylates (methyl-methacrylate (MMA), ethyl-methacrylate (EMA) and butyl-methacrylate (BMA)), tri(ethylene glycole) dimethacrylate, the synthesized ligand monomer and metal salts (zinc(II) or cobalt(II) acetate) enabling a shape-memory effect of the resulting dual networks.

Therefore, the current study focuses on the design of shape-memory metallopolymers and the elucidation of structure-property relationships. For this purpose, the temporary shape should be fixed via reversible metal ligand interactions. As ligand for the current system, a triazole-pyridine was chosen, which has not been used, as far as we know, in shape-memory metallopolymers. Furthermore, different methacrylates and a dimethacrylate (tri(ethylene glycol) dimethacrylate) as permanent crosslinker were utilized for the polymer synthesis (Figure 1). Additionally, these polymer networks also featured the pyridine–triazole ligands for the fixation of the temporary shape by the reversible formation of complexes with either zinc(II) or cobalt(II) acetate. The binding behavior of the metals to the ligands was studied using isothermal titration calorimetry (ITC) as well as spectroscopic methods like IR or Raman spectroscopy. Finally, the shape-memory behavior was investigated in detail and even triple-shape memory behavior could be obtained. The main goal of our work was to investigate the shape-memory process and to elucidate structure-property-relationships of our new metallopolymer networks based on a bis(triazole-pyridine) metal complex.

## 2. Experimental Part

### 2.1. Materials and Methods

All chemicals were used as received from TCI (Eschborn, Germany), Sigma Aldrich (Darmstadt, Germany), Alfa Aesar (Kandel, Germany), Thermo Fisher Scientific (Geel, Belgium) and Acros Organics (Geel, Belgium) if not otherwise stated. All solvents were dried over molecular sieve under nitrogen atmosphere. The used liquid monomers, methyl-methacrylate, ethyl-methacrylate, butyl-methacrylate, and tri(ethylene glycol) dimethacrylate, were destabilized over a short AlOx column (neutral AlOx, obtained from Molecula, Darlington, UK). 

Differential scanning calorimetry (DSC) was measured on a Netzsch DSC 204 F1 Phoenix instrument (Selb, Germany) under a nitrogen atmosphere with a heating rate of 10 and 20 K min^−1^. The thermogravimetric analysis (TGA) was carried under normal atmosphere using a Netzsch TG 209 F1 Iris (Selb, Germany).

Nuclear magnetic resonance spectra were measured using a Bruker AC 250 (250 MHz), Bruker AC 300 (300 MHz), Bruker AC 400 (400 MHz) and a Bruker AC 600 (600 MHz) spectrometers at 298 K (Billerica, MA, USA) if not stated differently. The chemical shift is given in parts per million (ppm on *δ* Scale) related to deuterated solvent. 

Elemental analysis was performed utilizing a Vario El III (Elementar, Langenselbold, Germany). 

FT-Raman spectra were recorded up to 4000 cm^−1^ with a spectral resolution of 4 cm^−1^ using a commercial Bruker MultiSpec spectrometer (Billerica, MA, United States). The Raman excitation light at 1064 nm was provided by a Nd:YAG laser (Klastech DeniCAFC-LC-3/40, Dortmund, Germany). The laser power at the samples was 1000 mW. The FT-Raman spectra were recorded using the software package OPUS 6.5. To analyze the temperature-dependent behavior of P12-Zn, temperature-dependent Raman spectra were recorded. The samples were heated via temperature controlled stage LTS 350 (Linkam Scientific, Tadworth, United Kingdom) with a heating rate of 1 °C/min. Five Raman spectra were recorded at 27 °C before the heating to 150 °C was started. A Raman spectrum consisting of 32 single scans was recorded every minute during the heating process. The raw Raman spectra were pre-processed using R (3.5.1). First the Raman spectra were restricted to the wavenumber region of interest, i.e., the region between 400 and 3200 cm^−1^. Subsequently, the Raman spectra were background-corrected using a SNIP algorithm (iterations = 50, order = 2, smoothing window = 3) and normalized to the CH-stretching area (2800 to 3100 cm^−1^).

Fourier transform infrared (FT-IR) spectra were recorded from 600 up to 4000 cm^−1^ using an IR-Affinity 1 (Shimadzu, Kyōto, Japan).

The cyclo-mechanic-tests were performed on a MCR 301 rheometer from Anton Paar (Graz, Austria) using the convection oven device CTD 450, which covers a broad temperature range from −150 to 450 °C. The samples were measured with a solid rectangular fixture setup (SRF12-SN13529, Anton Paar (Graz, Austria) in dimensions of approximately 29 × 10 mm (length, width) and a thickness of approx. 1.5–3.5 mm, the resulting sample gap was set to 15 mm. After fixing the sample, the temperature was set to 90 °C (respectively 110 °C). Subsequently, the sample was turned in a linear ramp (10°/min) until a shear stress of 18000 Pa was reached. In the following cooling step down to 25 °C (2 °C/min), the shear stress of 18000 Pa was kept constant. Afterwards, the shear stress was reduced to 0 Pa with a rate of 20 Pa/s. Finally, the sample was again heated to the initial temperature of 90 °C (respectively 110 °C; 2 °C/min). For operating the rheometer and analysis, the software RheoCompass^TM^ V1.24.549-Release 64-bit (Anton Paar, Graz, Austria) was used and the obtained results were exported as csv-files and further processed with OriginPro 2018 (OriginLab Corporation, Northampton, MA, USA).

### 2.2. Synthesis of the Monomer and Model System

#### 2.2.1. 11-[4-(Pyridine-2-yl)-1H-1,2,3-triazol-1-yl]undecane-1-ol (1)

The synthesis was performed according to a literature procedure [51]. A suspension of 11-bromundecan-1-ol (1.406 g, 5.60 mmol), sodium azide (390 mg, 5.60 mmol), copper sulfate pentahydrate (70 mg, 0.28 mmol), sodium ascorbate (278 mg, 1.40 mmol) and 2-ethynyl pyridine (580 mg, 5.63 mmol) in a ethanol/water-mixture (V: 7:3, 15 mL) was heated in the microwave to 125 °C for 25 min. Subsequently, a second addition of copper sulfate pentahydrate (70 mg, 0.28 mmol) occurred and the solution additionally was heated for 25 min at 125 °C in the microwave. A gel filtration (silica, ethyl acetate) provided the crude product as yellow solid, which was dissolved in dichloromethane. Precipitation from pentane yields **1** as withe powder (1.423 g, 80%).

^1^H NMR (400 MHz, CDCl_3_, *δ*): 1.11–1.43 (m, 14H, CH_2_), 1.47–1.67 (m, 2H, O-CH_2_-CH_2_), 1.78–2.05 (m, 3H, N–CH_2_–CH_2_, O*H*), 3.64 (t, *J* = 6.6 Hz, 2H, O–CH_2_), 4.42 (t, *J* = 7.1 Hz, 2H, N–CH_2_), 7.24 (dd, *J* = 6.8, 5.3 Hz, 1H), 7.80 (td, *J* = 7.8, 1.6 Hz, 1H), 7.97–8.26 (m, 2H), 8.58 (d, *J* = 4.4 Hz, 1H) ppm.

#### 2.2.2. 11-(4-(Pyridine-2-yl)-1H-1,2,3-triazol-1-yl)undecanyl-methacrylate (2)

The synthesis was performed under inert conditions and according to a literature report [52]. The 11-(4-(pyridin-2-yl)-1*H*-1,2,3-triazol-1-yl)undecan-1-ol (1) (4.00 g, 12.64 mmol) was dissolved in dry dichlormethane (60 mL). Triethylamine (3.37 mL, 42.28 mmol) was added. Subsequently, the solution was cooled to 0 °C and methacryloyl chloride (1.85 mL, 18.96 mmol) was added. The solution was warmed to room temperature and was stirred for additional 24 h. Afterwards, the reaction mixture was washed twice with saturated NaHCO_3_ solution. The organic layer was dried over Na_2_SO_4_ and the solvent was removed in vacuo, yielding a solid brown residue. Recrystallization from ethanol yields **2** as withe powder (4.18 g, 86%). 

^1^H NMR (300 MHz, CDCl_3_, *δ*): 1.17–1.43 (m, 14H, CH_2_), 1.63 (q, *J* = 6.9 Hz, 2H, O–CH_2_–CH_2_), 1.85–2.00 (m, 5H, N–CH_2_–C*H*_2_, CH_3_), 4.12 (t, *J* = 6.7 Hz, 2H, O–CH_2_), 4.40 (t, *J* = 7.2 Hz, 2H, N–CH_2_), 5.53 (s, 1H, =C*H*_2_), 6.08 (s, 1H, =CH_2_), 7.21 (dd, *J* = 7.5, 4.9 Hz, 1H), 7.76 (td, *J* = 7.8, 1.7 Hz, 1H), 8.05–8.29 (m, 2H), 8.57 (d, *J* = 4.8 Hz, 1H) ppm; ^13^C NMR (75 MHz, CDCl_3_, *δ*): 18.5 (*C*H_3_), 26.1 (*C*H_2_), 26.6 (*C*H_2_), 28.7 (*C*H_2_), 29.1 (*C*H_2_), 29.3 (*C*H_2_), 29.4 (*C*H_2_), 29.5 (*C*H_2_), 29.6 (*C*H_2_), 30.3 (*C*H_2_), 50.6 (N–*C*H_2_), 64.9 (O–*C*H_2_), 120.3, 121.9, 122.9, 125.3, 136.6, 136.7 148.4, 149.4, 150.5, 167.7 ppm; FT-IR (cm^−1^): 652, 788, 854, 930, 1045, 1076, 1161, 1300, 1323, 1462, 1597, 1640, 1717, 2847, 2920, 3129; C_22_H_32_N_4_O_2_: Calcd. C 68.72, H 8.39, N 14.57; found: C 68.57, H 8.41, N 14.31. 

#### 2.2.3. 11-(4-(Pyridine-2-yl)-1H-1,2,3-triazol-1-yl)undecanyl-acetate (3)

The synthesis and purification of 3 was performed analogue to the synthesis of 2 [52]. Thus, 11-(4-pyridin-2-yl)-1*H*-1,2,3-triazol-1-yl)undecan-1-ol (1) (696 mg, 2.2 mmol), dry dichloromethane (15 mL) as solvent and triethylamine (0.55 mL, 4.0 mmol) were utilized. Instead of methacryloyl chloride, acetyl chloride (0.25 mL, 3.5 mmol) was applied for the synthesis. Recrystallization from ethanol yields **3** as withe powder (553, 69%).

^1^H NMR (300 MHz, CDCl_3_, *δ*): 1.16–1.45 (m, 14H, CH_2_), 1.48–1.74 (m, 2H, O–CH_2_–CH_2_), 1.79 – 1.97 (m, 2H, N–CH_2_–CH_2_), 2.03 (s, 3H, CH_3_), 4.03 (t, *J* = 6.6 Hz, 2H, O–CH_2_), 4.41 (t, *J* = 7.0 Hz, 2H, N–CH_2_), 7.16–7.33 (m, 1H), 7.77 (t, *J* = 7.7 Hz, 1H), 8.04–8.27 (m, 2H), 8.57 (d, *J* = 3.8 Hz, 1H) ppm; ^13^C NMR (75 MHz, CDCl_3_, *δ*): 21.2 (*C*H_3_), 26.0 (*C*H_2_), 26.6 (*C*H_2_), 28.7 (*C*H_2_), 29.1 (*C*H_2_), 29.3 (*C*H_2_), 29.4 (*C*H_2_), 29.5 (*C*H_2_), 29.6 (*C*H_2_), 30.4 (*C*H_2_), 50.6 (N–*C*H_2_), 64.8 (O–*C*H_2_), 120.3, 121.9, 122.9, 137.0, 148.5, 149.5, 150.5, 171.4 ppm; C_20_H_30_N_4_O_2_: Calcd. C 67.01, H 8.44, N 15.63; found: C 66.78, H 8.32 N 15.44.

### 2.3. Synthesis of Model Complexes

The 11-(4-(pyridine-2-yl)-1H-1,2,3-triazol-1-yl)undecanyl-methacrylate (2) was dissolved in methanol (3 mL). M(OAc)_2_(H_2_O)x, dissolved in methanol (2 mL), was added to the ligand solution. The mixture turned to a clear and colored solution (zinc(II) acetate: light yellow; cobalt(II) acetate: purple). Afterwards the solvent was evaporated in vacuo yielding the resulting model complex ([Trpy_2_Zn]^2+^ and [Trpy_2_Co]^2+^). The utilized masses are summarized in Table 1.

[Trpy_2_Zn]^2+^: ^1^H NMR (300 MHz, CDCl_3_, *δ*): 1.02–1.46 (m, 30H, C*H*_2_), 1.56–1.79 (m, 4H, O–CH_2_–C*H*_2_), 1.85–2.00 (m, 18H, N–CH_2_–C*H*_2_, C*H*_3_), 4.14 (t, *J* = 6.6 Hz, 4H, O–C*H*_2_), 4.44 (t, *J* = 7.2 Hz, 4H, N–C*H*_2_), 5.56 (s, 2H, =C*H*_2_), 6.10 (s, 2H, =C*H*_2_), 7.33 (s, 2H), 7.86 (t, *J* = 7.5 Hz, 2H), 8.06–8.19 (m, 4H), 8.71 (s, 2H) ppm; FT-IR (cm^−1^): 617, 667, 787, 845, 930, 1045, 1161, 1296, 1323, 1415, 1454, 1597, 1717, 2851, 2920, 3129.

[Trpy_2_Co]^2+^: FT-IR (cm^−1^): 606, 644, 788, 852, 930, 1007, 1037, 1165, 1296, 1327, 1385, 1454, 1593, 1716, 2855, 2924, 3129.

### 2.4. Synthesis of the Polymer Networks

All polymerizations were performed in a 50 mL one-neck round bottom flask. A stock solution of the initiator, 2,2’-azobis(2-methylpropionitril) (AIBN), in dry *N*,*N*-dimethylformamide (DMF) was prepared. Compound 2, the respective monomer (methyl-methacrylate (MMA) in case of P1 to P5, ethyl-methacrylate (EMA) in case of P6 to P10 or butyl-methacrylate (BMA) in case of P10 to P13 and the crosslinker (triethylene glycol dimethacrylate (TEGDMA)) were added into the flask. Subsequently, the required amount of the initiator stock solution was added. The monomer-to-initiator ratio for all polymerizations was 100 to 1. Afterwards the residual volume of DMF was added to reach the desired concentrations of 1 M (P1 and P2) or 2 M (P3 to P13), respectively. The solution was purged with nitrogen for 45 min. Subsequently, the mixture was stirred in a preheated oil bath at 70 °C overnight. At the end of the polymerization the crude product was purified by the usage of dialysis (MWCO: 1000 g/mol, THF). The solvent was changed two times each day for three days. The respective used quantities of all substances are listed in Tables S1, S3 and S5. The ^1^H NMR spectra of the polymer networks (P1 to P13) are shown in the supporting information (SI). The results of the of the elemental analysis and the DSC and TGA investigations are shown in Tables S2, S4 and S6. All analytics of the polymers are summarized in the SI.

### 2.5. Synthesis of the Metallo Polymer Networks

For the synthesis of the metallopolymer networks (P1-Zn to P13-Zn and P1-Co to P13-Co) the polymers (P1 to P13) were swollen in chloroform (6 mL) resulting in gels. The calculated amount of either zinc(II) acetate or cobalt(II) acetate was dissolved in methanol (2 mL) and was added to the swollen polymer network. Afterwards, the solvent was evaporated. The resulting metallopolymer network was dried in vacuo at 40 °C and after that for one day at 80 to 90 °C in an oven. The respective used quantities of the substances are listed in Tables S7, S9 and S11. The results of the elemental analysis and the DSC and TGA investigations are shown in Tables S8, S10 and S12. All analytics of the polymers are summarized in the SI.

## 3. Results and Discussion

### 3.1. Synthesis of the Triazole-Pyridine Monomer and Model System

**Scheme 1:**
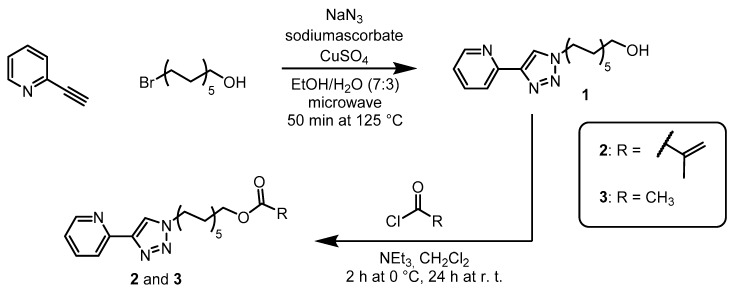
Schematic representation of the synthesis of 11-(4-(pyridin-2-yl)1*H*-1,2,3-triazol-1-yl)undecanyl-methacrylate (2) and 11-(4-(pyridin-2-yl)-1*H*-1,2,3-triazol-1-yl)undecanyl-acetate (3). [52] First step: synthesis of 11-(4-(pyridin-2-yl)-1*H*-1,2,3-triazol-1-yl)undecan-1-ol (1) via a cooper catalyzed 1,3-dipolar cycloaddition. Second step: Esterification of 11-(4-(pyridin-2-yl)-1*H*1,2,3-triazol-1-yl)undecan-1-ol with acetyl chloride or methacryloyl chloride.

In order to later synthesize polymers, the preparation of ligand-containing monomer was required. Furthermore, a low molar mass model system, mimicking the ligand in the polymer enabling the investigation of the complex formation, is useful. The synthesis of this model-system and the monomer was performed by a two-step reaction, which is depicted in Scheme 1. First, 11-(4-(pyridin-2-yl)-1*H*-1,2,3-triazol-1-yl)undecan-1-ol was prepared applying a copper catalyzed 1,3-dipolar cycloaddition of 11-bromo-1-undecanol, 2-ethynylpyridine and sodium azide [51]. Afterwards, an esterification was performed using triethylamine as base to shift the equilibrium towards the product side. As electrophile, methacryloyl chloride (in case of the monomer 2) and acetyl chloride (in case of the model system 3) were utilized. The reaction for the monomer is also reported in the literature [52] and was adapted for the model system. The products were characterized with ^1^H and ^13^C NMR spectroscopy as well as elemental analysis. The spectra are shown in the supporting information (Figures S1 to S4).

### 3.2. Isothermal Titration Calorimetry Investigations (ITC)

The synthesized model system (11-(4-[pyridin-2-yl]-1*H*-1,2,3-triazol-1-yl)undecanyl-acetate) was utilized for ITC investigations in order to determine the stoichiometry (L_n_M) of ligand to different metal ions as well as the complex association constant (K_a_). Furthermore, the complex dissociation constant (K_d_) can be determined, which corresponds to K_a_^−1^. These values are of great interest in order to evaluate the strength of the supramolecular unit and to correlate these molecular properties with the resulting properties of the material.

For the measurement, the salt was dissolved in methanol and put into the cell. The model system (3) was also dissolved in methanol and titrated to the solution of the salt at 303 K. Different salts have been investigated. Cobalt(II) acetate and zinc(II) acetate revealed good results (i.e., sufficient binding with a relatively low binding constant) as switching unit for shape-memory polymers, which are summarized in Table 2 (the ITC curves of the measurements are shown in the supporting information Figures S5 and S6). 

These results show that both metal salts are able to form complexes with the synthesized ligand. Therefore, two ligands bind one metal ion. The association constant for the cobalt(II) complex is higher than of the zinc(II) complex. Consequently, the cobalt(II) complex seems to be more stable than the formed zinc(II) complex. Generally, the complexation constants are relatively low compared to other metal complexes such as terpyridine [53]. Therefore, these complexes seems to be well-suited for the utilization within shape-memory polymers, since the metal–ligand interaction can easily be addressed by an external stimulus and the supramolecular unit can represent the reversible part of a shape-memory polymer. 

### 3.3. Model Complexes

**Scheme 2:**
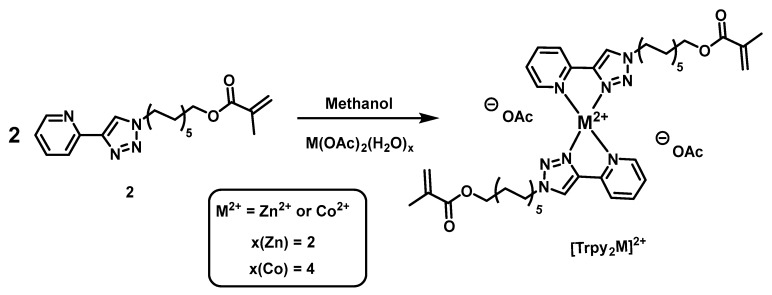
Schematic representation of the synthesis of the model complexes [Trpy_2_Zn]^2+^ and [Trpy_2_Co]^2+^.

According to the ITC-results, two model metal complexes were synthesized in order to analyze the metallopolymers later in a more detailed fashion. By this manner it is possible to compare the spectra of the model complexes with those of the metallopolymer networks, e.g., from NMR or FT-IR spectroscopy. Therefore, monomer (2) was dissolved in methanol and zinc(II) acetate dihydrate or cobalt(II) acetate tetrahydrate, respectively, was added. The ratio obtained from ITC was utilized for the complex synthesis. Evaporating the solvent yields the complexes [Trpy_2_Zn]^2+^ and [Trpy_2_Co]^2+^. The reaction for the synthesis of the model complexes is depicted in Scheme 2.

**Figure 2:**
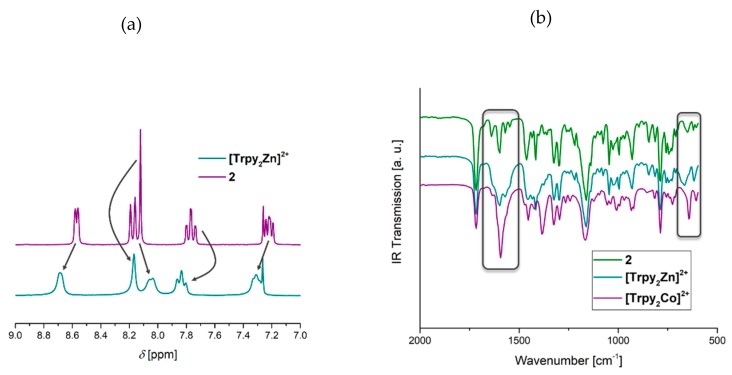
(**a**) Zoom of the ^1^H NMR spectra of 2 and the model complex containing zinc(II) ([Trpy_2_Zn]^2+^) (300 MHz, CDCl_3_); (**b**) zoom of the FT-IR spectra of 2 and the model complexes ([Trpy_2_ Zn]^2+^ and [Trpy_2_Co]^2+^).

The obtained complexes ([Trpy_2_Zn]^2+^ and [Trpy_2_Co]^2+^) were subsequently investigated using FT-IR spectroscopy and in the case of the zinc(II) containing complex by ^1^H NMR spectroscopy. The resulting spectra were compared with those of the monomer (2). A zoom of the ^1^H NMR spectra of 2 and [Trpy_2_Zn]^2+^ is shown in Figure 2a. By comparing both spectra, it can be seen that the complexation leads to a shift of the ligand signals (from 7.24 to 7.33 ppm; from 7.77 to 7.86 ppm and from 8.57 to 8.71 ppm) in the aromatic area within the ^1^H NMR spectrum. The FT-IR spectra of 2 and the model complexes ([Trpy_2_Zn]^2+^ and [Trpy_2_Co]^2+^) are shown in Figure 2b. An increase of the C=N valence vibration at 1597 cm^−1^ can be found for both complexes. Furthermore, some signals in the finger print area are shifted about a few cm^−1^. Thus, a successful complex formation can be visualized using IR spectroscopy. 

### 3.4. Polymer Networks

All polymer networks (P1 to P13) were synthesized via free radical polymerization (FRP). Azobis(isobutyronitrile) (AIBN) was utilized as initiator. As monomers, methyl-, ethyl- or butyl methacrylate were applied. Furthermore, a crosslinker, TEGDMA, as well as the synthesized ligand monomer 2, were used. The amounts of the ligand and the crosslinker were varied. The polymer networks feature a different crosslinking density (5% and 10%) and a different content of the ligand monomer (5%, 10% and 20%) in order to evaluate both influences later on. After the polymerization, none of the polymer networks was soluble in any common solvent. It was just possible to form gels by using for example chloroform, dichloromethane, DMF and THF. Thus, a successful formation of a polymer network can be assumed.

**Figure 3:**
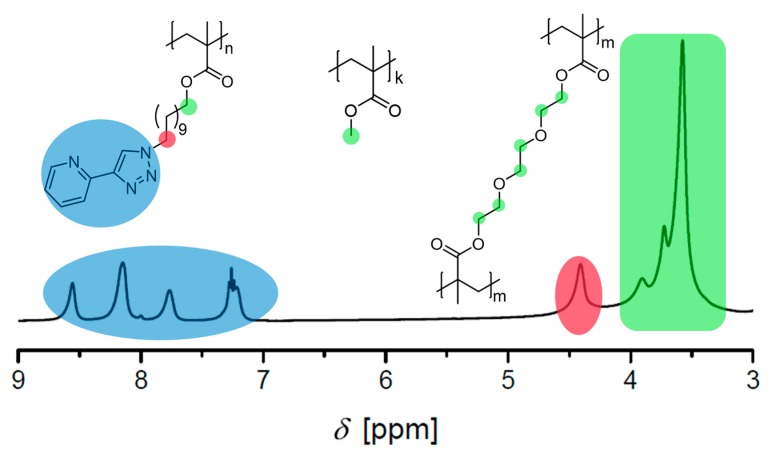
Zoom of the ^1^H NMR spectrum of the polymer network P3 as example for the determination of the ratio of MMA to the crosslinker (triethylene glycol dimethacrylate (TEGDMA)) and the ligand monomer. Blue and red: signals of the ligand, green: the signals of the O–CH_2_ and O–CH_3_ groups (400 MHz, CDCl_3_).

All resulting polymers were characterized by ^1^H NMR spectroscopy (see Figures S33 to S35) in the gel state and elemental analysis. These two methods were used to determine the ratio of the alkyl methacrylate to the ligand moieties assuming that the proportion of the crosslinker corresponds to the theoretical one. For NMR spectroscopy, the insoluble polymer networks were swollen in the NMR tube by adding deuterated chloroform. The theoretical proportion of the ligand units and the crosslinker in the polymer were assumed as fixed values and just the corresponding alkyl methacrylate content was determined. In the case of the NMR spectrum the ligand signals between 7.16 and 8.58 ppm were set in relation to those of the O–CH_3_ group of MMA (3 to 4 ppm), to the O–CH_2_ group in the case of EMA and BMA (3 to 5 ppm), respectively. It was important to note that the O–CH_2_ groups of the crosslinker and ligand monomer appear in the same range, so these protons had to be considered as well. In the case of EMA and BMA as main monomer, the signal of the O-CH_2_ groups gets broader and also the N-CH_2_ group at 4.41 ppm of the ligand monomer was not further base line separated and these protons has also to be considered. A typical spectrum of a polymer NMR is shown in Figure 3.

The calculation of the composition by the usage of the elemental analysis was performed using the determined values for the nitrogen. The theoretical proportion of the ligand units and the crosslinker in the polymer were assumed as fixed values and just the corresponding alkyl methacrylate content was determined. The initiator was neglected for the calculations.

The compositions of all polymer networks (P1 to P13) have been calculated in the same way and are summarized in Table 3. All found values for the amount of the main monomer in the polymer networks fit well to the theoretical expected values. However, both methods feature a certain limitation for the determination of the exact composition of polymer networks. In both cases it is not possible to determine the amount of cross-linker, consequently, the content must be assumed as fixed by the theoretical values. Nevertheless, the approximate ratio of the ligand to the main monomer could be revealed for nearly all polymers. 

### 3.5. Metallopolymer Networks

For the synthesis of the metallopolymer networks, the polymer networks (P1 to P13) were mixed with the corresponding metal salts. This reaction leads to supramolecular crosslinking by the formation of the ligand metal ion complex. As metal salts, zinc(II) acetate dihydrate (P1-Zn to P13-Zn) and cobalt(II) acetate tetrahydrate (P1-Co to P13-Co) were utilized. For the formation of the complex, the polymer network was swollen in chloroform. The metal salt was dissolved in methanol and added to the swollen polymer. After evaporation of the solvent, the metallopolymer networks (P1-Zn/Co to P13-Zn/Co) were obtained. The amount of metal salt was calculated based on the results of the ITC investigations. Thus, a metal–ligand ratio of 1:2 was applied for the synthesis of the supramolecular networks.

**Figure 4:**
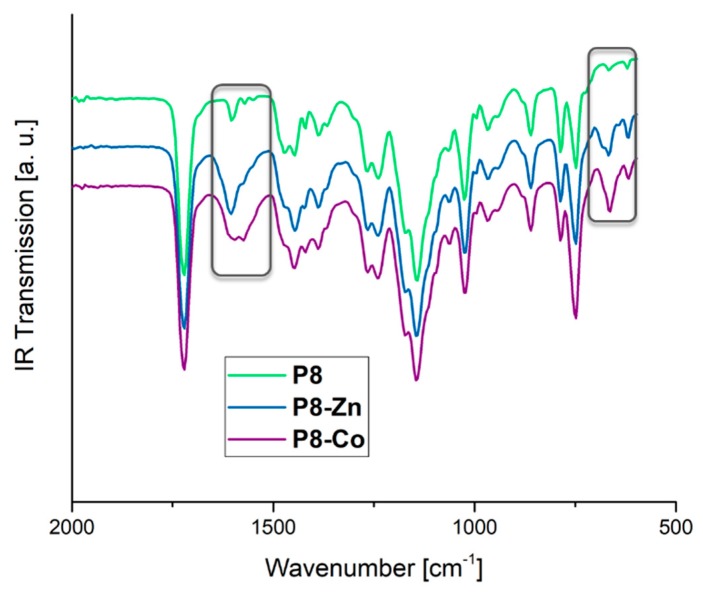
FT-IR spectra of the polymer network (P8) and the corresponding metallopolymer networks (P8-Zn and P8-Co).

The successful formation of the metallopolymer networks was verified via FT-IR and ^1^H NMR spectroscopy analogous to the model complexes. Exemplarily, the FT-IR spectra of the polymer network P8 and the corresponding metallopolymer networks (P8-Zn and P8-Co) are depicted in Figure 4. The largest change could be revealed for the N=C valence vibration at 1605 cm^−1^, which increases during complexation similar to the model complexes mentioned above. The signal in the fingerprint region at about 664 cm^−1^ shows an increase in intensity after metallopolymer formation: They revealed the same changes as found for the monomer (2) and the model complexes ([Trpy_2_Zn]^2+^ and [Trpy_2_Co]^2+^). These variations in the IR-spectra indicate a successful complex formation within the polymer networks resulting in a dual-polymer network structure featuring, on the one hand, an irreversible covalent and, on the other hand, a reversible supramolecular unit. Furthermore, the IR spectra were recorded and compared for all polymers and metallopolymer networks, which is depicted in the supporting information (Figures S37 to S49).

Exemplarily, the ^1^H NMR spectrum was recorded for the metallopolymer network P10-Zn, which contains zinc(II) ions. For this purpose, P10-Zn was swollen in the NMR tube by adding deuterated chloroform and the spectrum was obtained from the gel of the metallopolymer network. The zoom of the ^1^H NMR spectra of the polymer network P10 and the corresponding metallopolymer network P10-Zn is shown in the SI in Figure S36. Thus, a shift of the aromatic signals similar to the found shifts of the model complexes could be revealed indicating a successful complexation. However, further NMR-spectra of the other metallopolymers could not be obtained due the very low solubility as well as, in case of cobalt-based polymers, the paramagnetic behavior.

### 3.6. Thermal Behaviour of the Polymer and Metallopolymer Networks

The thermal behavior of the polymer networks P1 to P13 and the metallopolymer networks (P1-Zn to P13-Zn and P1-Co to P13-Co) was investigated by DSC and thermogravimetric analysis (TGA). The glass transition temperatures (*T_g_*) could be obtained by DSC measurements. All polymer and metallopolymer networks show a very broad *T_g_*. The TGA measurement provided the values for the degradation temperatures. The values for the glass transition temperature (*T_g_*) and the degradation temperature (*T_d_*) (Tables S2, S4 and S6) as well as the DSC (Figures S7 to S19) and TGA curves (Figures S20 to S32) are summarized in the supporting information. The results of the DSC measurement show, as expected, that the glass transition temperatures of the polymer and metallopolymer networks decrease with the length of the alkyl chain of the ester in the methacrylate [54]. Consequently, the MMA containing polymers (P1 to P5) and corresponding metallopolymers (P1-Zn/Co to P5-Zn/Co) feature the highest glass transition temperatures, those consisting of BMA (P11 to P13, P11-Zn/Co to P13-Zn/Co) the lowest ones. 

**Figure 5:**
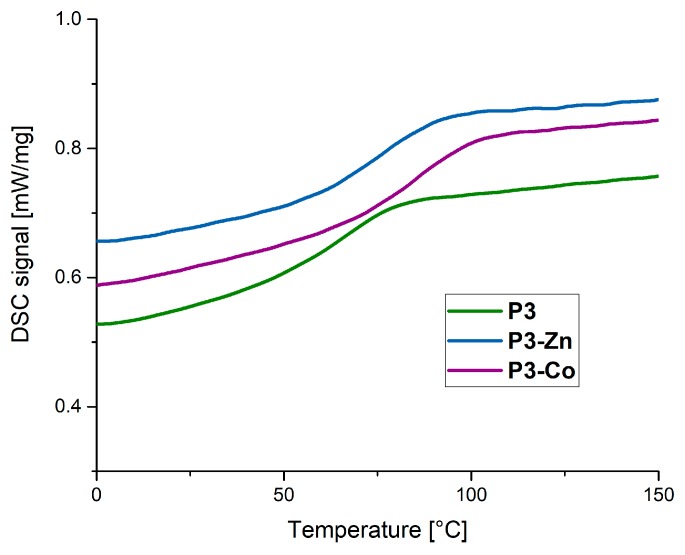
Differential scanning calorimetry (DSC) curves of the polymer network P3 (MMA, 5% TEGDMA, 20% Ligand, green) and the corresponding metallopolymer networks. Blue: P3-Zn (zinc (II) containing metallopolymer network). Purple: P3-Co (cobalt (II) containing metallopolymer network).

Besides the effect of the alkyl chain, there are also other trends that can be found. It turns out that the glass transition temperature of the polymer network is in general lower than these of the corresponding metallopolymer networks (Figure 5) indicating a successful complexation. Furthermore, nearly all of the investigated metal-containing polymers featured one very broad glass transition. Consequently, the activation of the metal-complexes of the metallopolymers cannot be detected by DSC or at least cannot be separated from the *T_g_*.

If both metallopolymers based on one polymer networks are compared, (Figure 5), it can be revealed that the glass transition temperature for the cobalt(II) ion containing metallopolymers is higher than that of the metallopolymer networks based on zinc(II) ions. These fit very well with the further ITC studies which showed a lower association constant for the zinc(II) containing complex.

An influence of the ligand content can also be observed. All polymers and metallopolymers with a higher amount of the ligand generally feature lower glass transition temperatures compared to those with a smaller amount of ligand monomer. This behavior can also be explained by the plasticizer effect of the long alkyl chain based ligand entity.

The TGA measurement revealed values for the degradation temperatures of the polymer and the metallopolymer networks. They were performed under normal atmosphere. All polymers are stable above 200 °C, most also above 250 °C. It could be observed that the cobalt(II) ion containing metallopolymer networks in general featured a slightly lower degradation temperature than the zinc(II) ion containing metallopolymer networks. 

Exemplarily, metallopolymer network P12-Zn was characterized by temperature-dependent Raman spectroscopy in order to reveal the temperature-dependent binding of the metal complexes. However, even up to 150 °C, no change in the Raman spectrum could be observed. The spectra are depicted in the supporting information in Figure S50. Consequently, no complete decomplexation can be revealed indicating that a potential dynamic behavior will be based on exchange reactions, which is typically found for vitrimeric systems [55]. Similar findings were previously also reported for other metallopolymer systems [56].

### 3.7. Shape-Memory Ability of the Metallopolymer Networks

**Figure 6:**
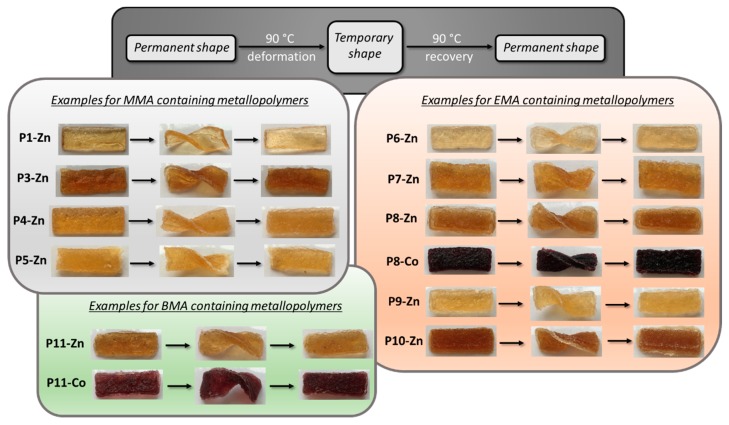
Schematic representation (grey) and photo series of the shape-memory test of different metallopolymer networks containing MMA (light grey), EMA (red) and BMA (green).

To investigate the shape-memory properties of the synthesized metallopolymer networks (P1-Zn to P13-Zn and P1-Co to P13-Co), a permanent shape had to be determined by an initial process. Therefore, the corresponding metallopolymer was pressed at 130 °C with a pressure between 1 to 4 t in a special mold. By this manner, small rectangular polymer samples could be fabricated. Consequently, the rectangular shape was the permanent shape (see Figure 6). Afterwards, the metallopolymer network, in its permanent shape, was heated up to 90 °C, twisted and cooled to room temperature. This was the programming step of the temporary shape. The metallopolymer network stayed in its new form until it was heated up again to 90 °C. 

During the pressing of the polymers it was found that in general the zinc(II) containing metallopolymer networks could be processed easier than the corresponding ones containing cobalt(II). For some metallopolymer networks, mostly those containing cobalt(II), it was not even possible to press the metallopolymer network into a smooth transparent specimen. In these cases, the samples were crumbly and brittle at room temperature. 

In general, nearly all metallopolymer networks featured shape-memory behavior, even those featuring an insufficient processing step. The shape recovery just took a few seconds at 90 °C. The polymers containing butyl methacrylate could be deformed the easiest followed by those consisting of ethyl methacrylate. Those based on methyl methacrylate were the hardest and it was difficult to deform them at 90 °C, which is caused by the high *T_g_* of the metallopolymer networks.

**Figure 7:**
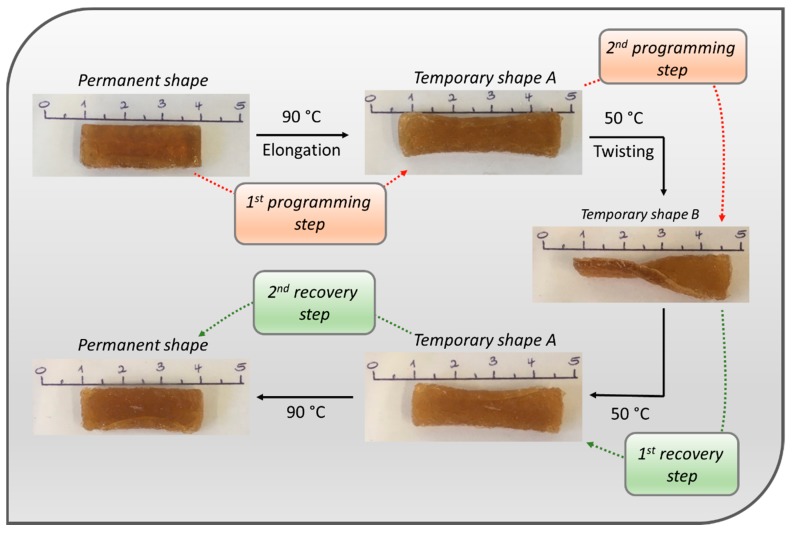
Photo series of the triple-shape-memory test of the metallopolymer network P12-Zn.

The metallopolymer network P12-Zn was chosen for testing of the triple-shape-memory effect, since it was very soft and flexible at 90 °C. Thus, it could be revealed that the polymer can fix more than one temporary shape (Figure 7). Therefore, it first was heated up to 90 °C, elongated at this temperature and after that cooled to room temperature. This elongated form is the temporary shape A. Subsequently, it was heated up to 50 °C, twisted at this temperature and cooled to room temperature. This elongated and twisted form is the temporary shape B. Heating the polymer up to 50 °C leads to the recovery of the temporary shape A, which was not twisted anymore but elongated. Heating the polymer again up to 90 °C leads to the recovery of the original permanent shape under shrinking. 

To determine the fixity and recovery rates of the synthesized metallopolymer networks and also to compare them regarding their different composition, selected samples were investigated using a cyclo-mechanic-tests. The metallopolymer networks P1-Zn, P6-Zn, P7-Zn, P8-Zn and P11-Zn were chosen for the test, to investigate the influence of the different compositions. P1-Zn, P6-Zn and P11-Zn all consist of 5% crosslinker and 5% ligand, just the main monomer is different, so these three measurements where considered as useful to compare the influence of the used main monomer. The metallopolymer networks P6-Zn, P7-Zn and P8-Zn just differ in the content of the ligand, so these three polymers were assumed to be helpful to investigate the influence of the ligand content on the shape-memory abilities. For each sample the measurement was performed three times. The strain fixity rate (*R_f_*) quantifies the ability to fix a mechanical deformation (*ε_m_*) and leads to the temporary shape (*ε_u_*) [20]. Furthermore, the strain recovery rate (*R_r_*), which quantifies the ability to restore the mechanical deformation of the permanent shape (*ε_p_*), can be determined. The test is usually repeated several times. The number of repeating corresponds to the number of cycles (*N*) [20]. The values for *R_f_* and *R_r_* can be calculated by Equations (1) and (2).

*R_f_* (*N*) = *ε_u_*(*N*)/*ε_m_*(*N*) × 100 (1)

*R_r_* (*N*) = (*ε_m_*(*N*) − *ε_p_*(*N*))/(*ε_m_*(*N*) − *ε_p_*(*N* − 1)) × 100. (2)

**Figure 8:**
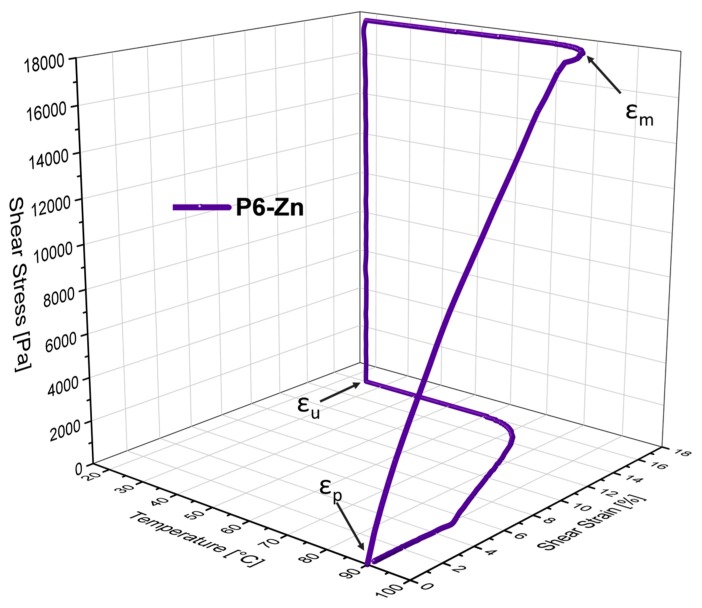
The 3D plot (temperature–stress–strain diagram) of the metallopolymer network P6-Zn.

For the measurement, all metallopolymer samples were heated to 90 °C (with the exception of the metallopolymer sample P1-Zn, which was heated to 110 °C). Subsequently, the sample was turned in a linear ramp until the shear stress reached 18,000 Pa, leading to the deformation of the sample. The shear stress was kept constant during the following cooling step of the sample. At a temperature of about 25 °C, the shear stress was reduced to 0 Pa (linear ramp, 20 Pa/s) and the sample was again heated to 90 °C (respectively 110 °C for P1-Zn). The resulting 3D plot (stress–strain-temperature diagram) of the first cycle of the sample P6-Zn is shown in Figure 8. The revealed values for *ε_p_*, *ε_m_*, *ε_u_*, as well as the plot of the first cycle, are summarized in the supporting information (Table S13, Figures S51 to S55) for all investigated metallopolymer networks. The calculated values for the fixity and recovery rates for the samples P1-Zn, P6-Zn, P7-Zn, P8-Zn and P11-Zn are shown in Table 4. It was found that nearly all investigated metallopolymer networks reveal very good fixity (above 98%) and recovery rates (above 95%) and also no loss of the shape-memory ability could be observed during the three cycles. Just the recovery rate of the metallopolymer network which consists of MMA was a bit lower. Beside this fact, no significant influence of the composition could be observed caused by the fact that nearly all investigated networks show a very good shape-memory performance. Just the switching temperature differs depending on the used main monomer. 

Comparing the herein presented system with already existing shape-memory metallopolymers, our fixity rate is in general higher. The 4-oxy-2,6-bis(*N*-methylbenzimidazolyl)pyridine-europium salt system from Kumpfer et al. showed a fixity rate between 87 to 90% [46]. The triphenylphosphine-Rh system, which also can be found in literature, just was able to fix around 60% of the deformation [48]. The recovery rates of these polymers were about 90% [46,48]. The herein presented triazole-pyridine-zinc system also shows in general slightly higher values.

**Figure 9:**
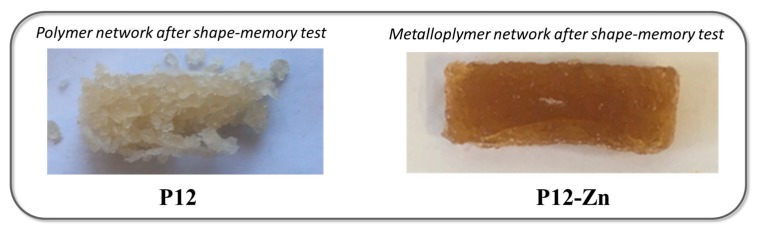
Photos of the polymer networks P12 (**left**) and the metallopolymer network P12-Zn (**right**) after the shape-memory test.

To investigate the influence of the formed bis(triazole-pyridine) complex on the shape-memory abilities, also the polymer network P12 (without any metal complex) was pressed at 120 °C in a rectangular piece and a shape-memory test was performed. The polymer piece was also heated to 90 °C and deformed. Heating the polymer again to 90 °C should subsequently trigger the recovery process, but the polymer piece turns very brittle during the recovery step and crumbled in several small pieces (see Figure 9). In opposite to this, the metallopolymer network P12-Zn, which was synthesized from this polymer network even featured triple-shape-memory behavior without losing its transparency or getting crumbly. Figure 9 shows the comparison between P12 and P12-Zn after the shape recovery.

## 4. Conclusions

In this work, we showed the successful synthesis of metallopolymer networks featuring a shape-memory effect. The switching unit was obtained by a bis(triazole-pyridine)-metal complex. The polymer networks were synthesized via free radical polymerization and the composition was calculated by elemental analysis and ^1^H NMR spectroscopy. As metal salt for the formation of the metallopolymer networks zinc(II) and cobalt(II) acetate was chosen, caused by the results of the ITC investigations. The thermal behavior of the polymer and the metallopolymer networks was investigated via DSC and TGA measurements and also the successful complexation in the metallopolymer networks was investigated via FT-IR spectroscopy and exemplarily via ^1^H NMR spectroscopy. Finally, shape-memory test showed good shape-memory abilities and even the fixation and selective recovery of two different temporary shapes (triple shape-memory) was possible. A performed cyclo-mechanic-test revealed very good fixity (above 98%) and recovery rates (above 95%) for all synthesized metallopolymer networks.

Future work will focus on a more detailed investigation of the ongoing molecular processes during the shape recovery process and an analysis of more metal complexes featuring other binding constants. Thus, the impact of the strength of the supramolecular bond on the shape-memory behavior will be analyzed in detail

## Figures and Tables

**Table 1:** Utilized masses for the synthesis of the model complexes ([Trpy_2_Zn]^2+^ and [Trpy_2_Co]^2+^).

	Educts	m [mg]	n [mmol]	Yield [mg]
**[Trpy_2_Zn]^2+^**	2 Zn(OAc)_2_ 2H_2_O	247 71	0.642 0.321	318 (100%)
**[Trpy_2_Co]^2+^**	2 Co(OAc)_2_ 4H_2_O	220 71	0.572 0.286	291 (100%)

**Table 2:** Determined complex dissociation and association constants and stoichiometry for zinc(II) and cobalt(II) acetate and the model system by titration calorimetry investigations (ITC) investigations.

Metal Salt	K_d_	K_a_	n
Zn(OAc)_2_	2.95 × 10^−3^	3.39 × 10^2^	1.8
Co(OAc)_2_	3.74 × 10^−4^	2.68 × 10^3^	2.4

**Table 3:** Theoretical and calculated values for the composition of the polymer networks P1 to P13.

	Main Monomer	Theoretical Units of the TEGDMA	Theoretical Units of the Ligand	Units of the Main Monomer (MMA, EMA or BMA)		
				Theoretical	Found by NMR	Found by EA
**P1**	**MMA**	**1**	1	20	18.3	14.6
**P2**		1	2	20	19.7	18.5
**P3**		1	4	20	20.3	22.0
**P4**		2	2	20	21.7	22.7
**P5**		2	4	20	16.7	21.1
**P6**	EMA	1	1	20	20.0	19.7
**P7**		1	2	20	16.0	16.1
**P8**		1	4	20	21.0	21.3
**P9**		2	2	20	18.0	18.2
**P10**		2	4	20	20.5	21.5
**P11**	BMA	1	1	20	21.0	21.6
**P12**		1	2	20	21.0	19.7
**P13**		2	2	20	19.5	21.0

**Table 4:** Calculated fixity and recovery rates of the metallopolymer networks P1-Zn, P6-Zn, P7-Zn, P8-Zn and P11-Zn via cyclo-mechanic-tests.

Sample	Cycle	*R_f_* [%]	*R_r_* [%]
P1-Zn (MMA, 5% crosslinker, 5% ligand)	**1**	99.8	84.4
	**2**	100	85.5
	**3**	100	81.8
	**Average**	99.9	83.9
P6-Zn (EMA, 5% crosslinker, 5% ligand)	**1**	98.7	95.1
	**2**	98.7	95.0
	**3**	98.7	96.2
	**Average**	98.7	95.5
P7-Zn (EMA, 5% crosslinker, 10% ligand)	**1**	98.5	95.2
	**2**	98.4	95.4
	**3**	98.4	95.4
	**Average**	98.4	95.3
P8-Zn (EMA, 5% crosslinker, 10% ligand)	**1**	98.5	96.3
	**2**	98.0	97.5
	**3**	98.0	98.2
	**Average**	98.2	97.3
P11-Zn (BMA, 5% crosslinker, 5% ligand)	**1**	99.1	96.6
	**2**	99.1	95.8
	**3**	100.0	96.6
	**Average**	99.4	96.3

## Supplementary Materials

The following are available online at https://www.mdpi.com/2073-4360/11/11/1889/s1, Figure S1 to S55, Table S1 to S13.
