# Supplementary material for: Shape-Memory Metallopolymer Networks Based on a Triazole–Pyridine Ligand
Source: Polymers (Basel). 2019 Nov 15;11(11):1889. doi: 10.3390/polym11111889 (PMC6918370; doi:10.3390/polym11111889)
Supplement: Supplementary file 1 [file polymers-11-01889-s001.pdf]

Supplementary information

Shape-memory metallopolymer networks based on a triazole-pyridine-ligand

Josefine Meurer [1,2], Julian Hniopek [3,4,5], Stefan Zechel [1,2], Marcel Enke [1,2], Jürgen Vitz [1,2], Michael Schmitt [3,4], Jürgen Popp [3,4,5], Martin D. Hager [1,2] and Ulrich S. Schubert [1,2,*]

[1] Laboratory of Organic and Macromolecular Chemistry (IOMC), Friedrich Schiller University Jena, Humboldtstr. 10, 07743 Jena, Germany; josefine.meurer@uni-jena.de (J.M.); stefan.zechel@uni-jena.de (S.Z.); marcel.enke@uni-jena.de (M.E.); martin.hager@uni-jena.de (M.D.H.)

[2] Jena Center of Soft Matter (JCSM), Friedrich Schiller University Jena, Philosophenweg 7, 07743 Jena, Germany

[3] Institute of Physical Chemistry (IPC), Friedrich Schiller University Jena, Helmholzweg 4, 07743 Jena, Germany; julian.hniopek@uni-jena.de (J.H.); m.schmitt@uni-jena.de (M.S.); juergen.popp@uni-jena.de (J.P.)

[4] Abbe Center of Photonics, Friedrich Schiller University Jena, Albert-Einstein-Straße 6, 07745 Jena, Germany

[5] Leibniz Institute of Photonic Technology, e. V. Jena, Albert-Einstein-Str. 9, 07745 Jena, Germany

[*] Correspondence: ulrich.schubert@uni-jena.de

Table of content

Characterization of the monomer (2), the model system (3) **2**

NMR spectra of the monomer (**2**) and the model system (**3**) 2

Isothermal titration calorimetry of 11-[4-(pyridine-2-yl)-1H-1,2,3-triazol-1-yl]undecanyl-acetate (3) **5**

Synthesis and characterization of the polymer networks (P1 to P13) and the metallopolymer networks (P1-Zn/Co to P13-Zn/Co) **6**

Differential scanning calorimetry of the polymer networks (**P1** to **P13**) and the metallopolymer networks (**P1-Zn/Co** to **P13-Zn/Co**) 10

Thermogravimetric analysis (TGA) of the polymer networks (**P1** to **P13**) and the metallo-polymer networks (**P1-Zn/Co** to **P13-Zn/Co**) 17

NMR spectra of the polymer networks (**P1** to **P13**) 24

IR spectroscopic investigation of the polymer (**P1** to **P13**) and metallo-polymer networks (**P1-Zn/Co** to **P13-Zn/Co**) 28

Temperature dependent Raman spectroscopy of **P12-Zn** 35

Cyclo-mechanic-tests of selected metallopolymer networks 36

Characterization of the Monomer (2), the Model System (3)

NMR Spectra of the Monomer (2) and the Model System (3)

Nuclear magnetic resonance spectra were measured using a Bruker AC 250 (250 MHz), Bruker AC 300 (300 MHz), Bruker AC 400 (400 MHz) and a Bruker AC 600 (600 MHz) spectrometers at 298 K if not stated differently. The chemical shift is given in parts per million (ppm on δ Scale) related to deuterated solvent.

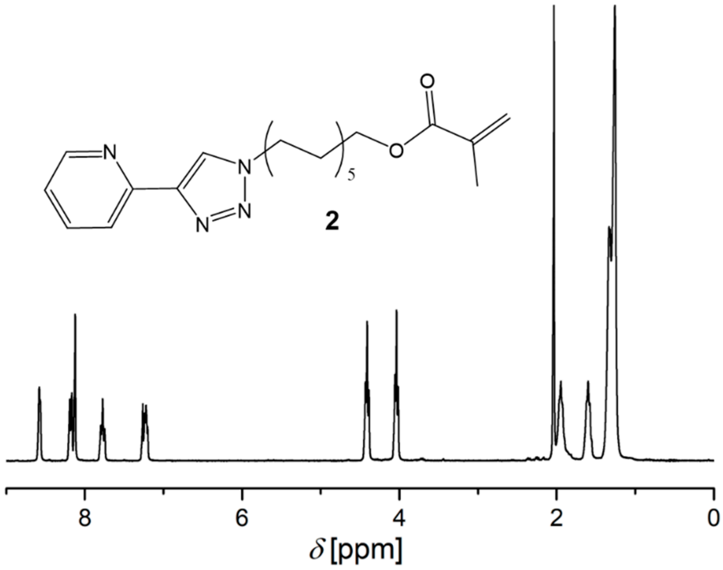

Figure S1. ^{1}H NMR spectrum of 11-[4-(pyridin-2-yl)-1*H*-1,2,3-triazol-1-yl]undecanyl-methacrylate (**2**) (300 MHz, $CDCl_3$).

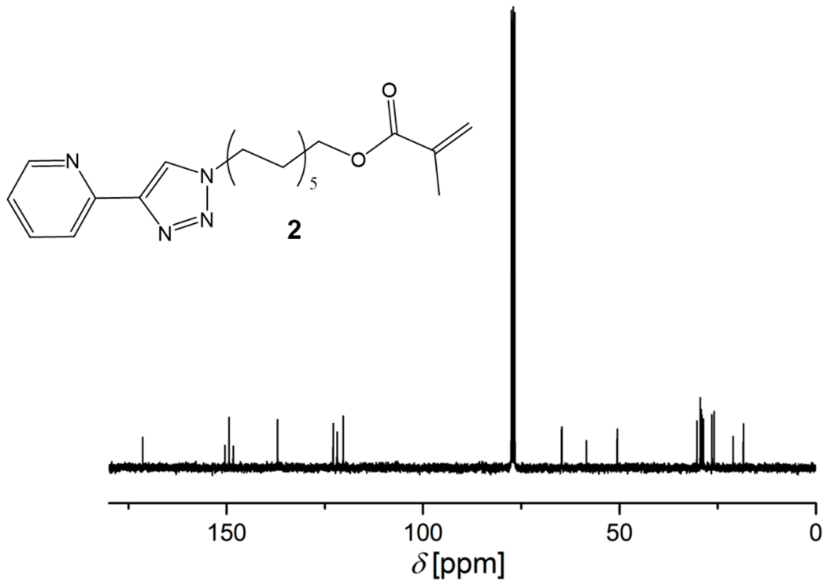

Figure S2. ^{1}H NMR spectrum of 11-[4-(pyridin-2-yl)-1*H*-1,2,3-triazol-1-yl]undecanyl-methacrylate (**2**) (75 MHz, $CDCl_3$).

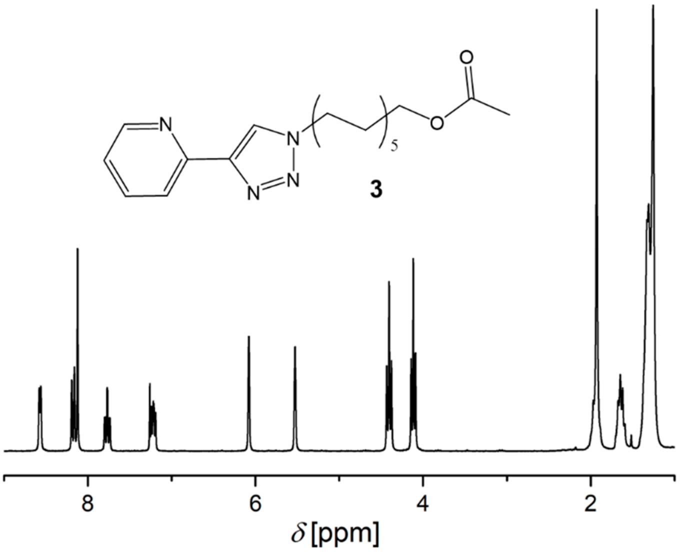

Figure S3. ^{1}H NMR spectrum of 11-[4-(pyridin-2-yl)-1*H*-1,2,3-triazol-1-yl]undecanyl-acetate (**3**) (300 MHz, $CDCl_3$).

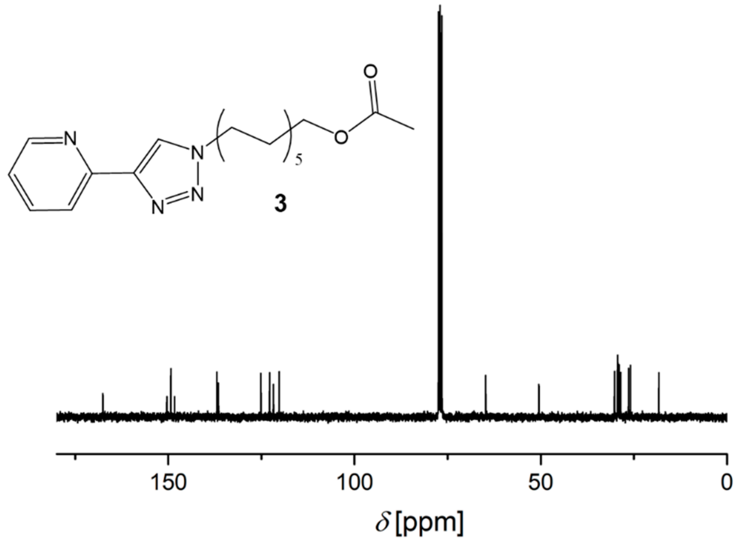

Figure S4. ^{13}C NMR spectrum of 11-[4-(pyridin-2-yl)-1*H*-1,2,3-triazol-1-yl]undecanyl-acetate (**3**) (75 MHz, $CDCl_3$).

Isothermal Titration Calorimetry of 11-[4-(pyridine-2-yl)-1H-1,2,3-triazol-1-yl]undecanyl-acetate (3)

All titrations were performed using a standard volume Nano ITC (TA Instruments) at 303 K. Solutions were always prepared prior to use in dry solvents using vacuum dried ligand and metal salt. Blank titrations in dry ligand were performed and subtracted from the corresponding titrations to remove the effect of dilution. The fitting of the measured data was performed with the NanoAnalyze program from TA instruments.

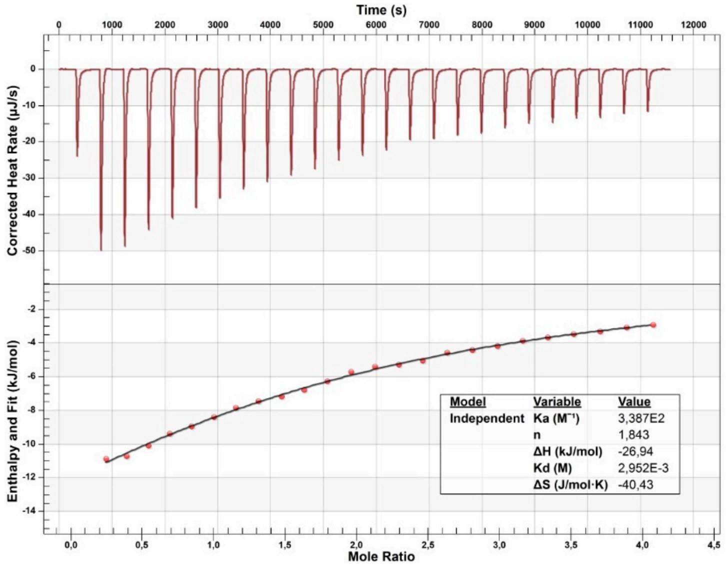

Figure S5. ITC titration data of $Zn(OAc)_2$ (1.25 mM, in cell) with **3** (17.10 mM, in syringe) in MeOH at 303 K.

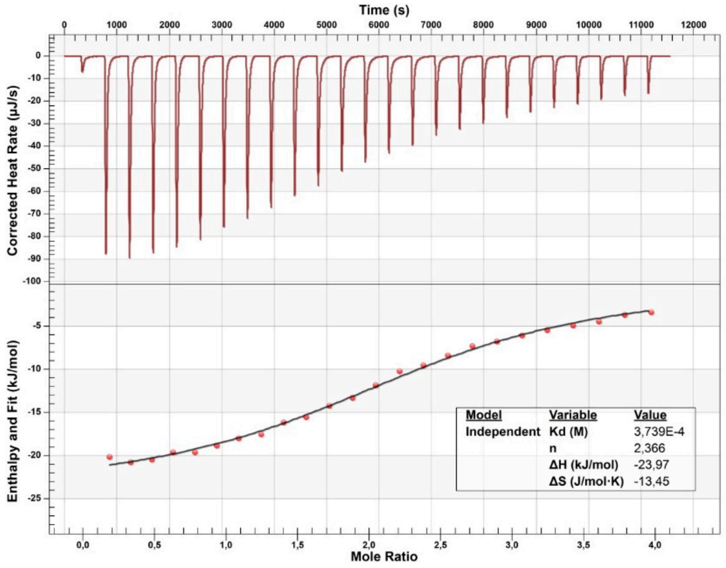

Figure S6. ITC titration data of $Co(OAc)_2$ (1.25 mM, in cell) with 3 (17.06 mM, in syringe) in MeOH at 303 K.

Synthesis and Characterization of the Polymer networks (P1 to P13) and the Metallopolymer Networks (P1-Zn/Co to P13-Zn/Co)

Table S1. Utilized masses and volumes for the copolymerization of the polymer networks containing MMA (**P1** to **P5**).

Polymer	Monomers	m [g] (monomer)	n [mmol] (monomer)	m [mg] (AIBN)	V [mL] (DMF)
	MMA	4.00	39.95		
**P1**	TEGDMA	0.57	1.99	72.17	41.95
	**2**	0.77	1.99		
	MMA	3.50	34.96		
**P2**	TEGDMA	0.50	1.75	66.02	36.71
	**2**	1.34	3.50		
	MMA	2.70	26.97		
**P3**	TEGDMA	0.39	1.35	55.34	14.16
	**2**	2.07	5.39		
	MMA	3.00	29.96		
**P4**	TEGDMA	0.86	2.99	59.04	16.48
	**2**	1.15	2.99		
	MMA	2.50	24.97		
**P5**	TEGDMA	0.72	2.50	53.30	13.73
	**2**	1.92	5.00		

Table S2. Results of the elemental analyses and the DSC and TGA investigations for the polymers containing MMA (**P1** to **P5**).

Polymer	Found in elemental analysis [%]			T_g [°C]		T_d [°C]
	C	H	N	Range	Middle	
**P1**	60.09	8.09	2.66	87 to 110	98	210
**P2**	60.45	7.95	3.85	61 to 92	77	230
**P3**	59.50	7.74	5.57	45 to 80	63	240
**P4**	56.90	7.52	3.10	69 to 106	87	220
**P5**	59.29	7.72	5.30	48 to 86	67	203

Table S3. Utilized masses and volumes for the copolymerization of the polymer networks containing EMA (**P6** to **P10**).

Polymer	Monomers	m [g] (monomer)	n [mmol] (monomer)	m [mg] (AIBN)	V [mL] (DMF)
	EMA	4.00	35.04		
**P6**	TEGDMA	0.50	1.75	63.30	18.40
	**2**	0.67	1.75		
	EMA	3.50	30.66		
**P7**	TEGDMA	0.44	1.53	57.91	16.10
	**2**	1.18	3.07		
	EMA	3.00	26.28		
**P8**	TEGDMA	0.38	1.310	53.95	13.80
	**2**	2.02	5.26		
	EMA	3.50	30.66		
**P9**	TEGDMA	0.88	3.07	60.42	16.87
	**2**	1.18	3.07		

	EMA	3.00	26.28		
**P10**	TEGDMA	0.75	2.63	56.11	14.46
	**2**	2.02	5.26		

Table S4. Results of the elemental analyses and the DSC and TGA investigations for the polymers containing MMA (**P6** to **P10**).

Polymer	Found in elemental analysis [%]			T_g [°C]		T_d [°C]
	C	H	N	Range	Middle	
**P6**	60.73	8.27	1.93	50 to 84	67	230
**P7**	61.46	8.39	3.89	48 to 75	62	225
**P8**	63.07	8.33	5.29	38 to 63	51	220
**P9**	58.53	8.06	3.30	53 to 83	68	210
**P10**	61.94	8.22	4.93	45 to 65	55	225

Table S5. Utilized masses and volumes for the copolymerization of the polymer networks containing BMA (**P11** to **P13**).

Polymer	Monomers	m [g] (monomer)	n [mmol] (monomer)	m [mg] (AIBN)	V [mL] (DMF)
	BMA	4.00	28.13		
**P11**	TEGDMA	0.40	1.41	50.81	14.77
	**2**	0.54	1.41		
	BMA	4.00	28.13		
**P12**	TEGDMA	0.40	1.41	53.12	14.77
	**2**	1.08	2.81		
	BMA	3.5	24.61		
**P13**	TEGDMA	0.705	2.46		13.34
	**2**	0.946	2.46		

Table S6. Results of the elemental analyses and the DSC and TGA investigations for the polymers containing MMA (**P11** to **P13**).

Polymer	Found in elemental analysis [%]			T_g [°C]		T_d [°C]
	C	H	N	Range	Middle	
**P11**	63.39	9.16	1.50	34 to 52	43	257
**P12**	65.46	9.45	2.91	22 to 49	35	260
**P13**	66.61	9.36	2.60	26 to 53	39	251

Table S7. Utilized masses for the synthesis of the metallopolymer networks containing MMA (**MP1** to **MP10**).

Metallo polymer	Polymer	m [mg] (polymer)	Metal salt	m [mg] (metal salt)
**P1-Zn**	**P1**	1469	$Zn(OAc)_2 \times 2\ H_2O$	60
**P1-Co**		1485	$Co(OAc)_2 \times 4\ H_2O$	69
**P2-Zn**	**P2**	609	$Zn(OAc)_2 \times 2\ H_2O$	44
**P2-Co**		1435	$Co(OAc)_2 \times 4\ H_2O$	116
**P3-Zn**	**P3**	1634	$Zn(OAc)_2 \times 2\ H_2O$	187
**P3-Co**		1493	$Co(OAc)_2 \times 4\ H_2O$	194
**P4-Zn**	**P4**	1592	$Zn(OAc)_2 \times 2\ H_2O$	104
**P4-Co**		1637	$Co(OAc)_2 \times 4\ H_2O$	122

**P5-Zn**	**P5**	1662	$Zn(OAc)_2 \times 2\ H_2O$	177
**P5-Co**		1639	$Co(OAc)_2 \times 4\ H_2O$	198

Table S8. Results of the elemental analyses and the DSC and TGA investigations for the metallopolymer networks containing MMA (**MP1** to **P10**).

Metallo polymer	Found in elemental analysis [%]			T_g [°C]		T_d [°C]
	C	H	N	Range	Middle	
**P1-Zn**	54.46	7.24	2.26	97 to 115	106	271
**P1-Co**	53.39	7.08	2.15	85 to 109	97	248
**P2-Zn**	57.20	7.47	3.57	82 to 103	92	257
**P2-Co**	55.46	7.28	3.40	81 to 105	93	256
**P3-Zn**	54.64	7.06	4.85	56 to 89	73	289
**P3-Co**	53.19	6.85	4.77	65 to 102	83	267
**P4-Zn**	54.67	7.15	3.00	59 to 106	83	277
**P4-Co**	55.63	7.30	3.16	70 to 106	88	250
**P5-Zn**	55.87	7.23	4.74	53 to 99	83	287
**P5-Co**	55.33	7.21	4.75	66 to 99	83	260

Table S9. Utilized masses for the synthesis of the metallopolymer networks containing EMA (**MP11** to **MP20**).

Metallo polymer	Polymer	m [mg] (polymer)	Metal salt	m [mg] (metal salt)
**P6-Zn**	**P6**	1500	$Zn(OAc)_2 \times 2\ H_2O$	56
**P6-Co**		1501	$Co(OAc)_2 \times 4\ H_2O$	63
**P7-Zn**	**P7**	1568	$Zn(OAc)_2 \times 2\ H_2O$	103
**P7-Co**		1650	$Co(OAc)_2 \times 4\ H_2O$	123
**P8-Zn**	**P8**	1488	$Zn(OAc)_2 \times 2\ H_2O$	159
**P8-Co**		1621	$Co(OAc)_2 \times 4\ H_2O$	197
**P9-Zn**	**P9**	1684	$Zn(OAc)_2 \times 2\ H_2O$	101
**P9-Co**		1495	$Co(OAc)_2 \times 4\ H_2O$	102
**P10-Zn**	**P10**	1629	$Zn(OAc)_2 \times 2\ H_2O$	163
**P10-Co**		1612	$Co(OAc)_2 \times 4\ H_2O$	183

Table S10. Results of the elemental analyses and the DSC and TGA investigations for the metallopolymer networks containing EMA (**MP11** to **P20**).

Metallo polymer	Found in elemental analysis [%]			T_g [°C]		T_d [°C]
	C	H	N	Range	Middle	
**P6-Zn**	59.55	8.16	1.90	48 to 78	68	212
**P6-Co**	59.13	8.12	1.83	59 to 99	79	257
**P7-Zn**	58.98	7.96	3.52	54 to 87	71	276
**P7-Co**	58.89	8.04	3.35	53 to 84	69	260
**P8-Zn**	58.94	7.81	4.77	49 to 80	65	269
**P8-Co**	58.58	7.78	4.66	52 to 96	74	265
**P9-Zn**	59.48	7.93	3.26	50 to 87	68	264
**P9-Co**	58.11	7.78	3.28	51 to 92	71	253
**P10-Zn**	58.27	7.71	4.74	44 to 80	62	285
**P10-Co**	58.01	7.63	4.36	53 to 94	73	251

Table S11. Utilized masses for the synthesis of the metallopolymer networks containing BMA (**MP21** to **P26**).

Metallo polymer	Polymer	m [mg] (polymer)	Metal salt	m [mg] (metal salt)
**P11-Zn**	**P11**	1506	$Zn(OAc)_2 \times 2\ H_2O$	47
**P11-Co**		1467	$Co(OAc)_2 \times 4\ H_2O$	51
**P12-Zn**	**P12**	1456	$Zn(OAc)_2 \times 2\ H_2O$	82
**P12-Co**		1496	$Co(OAc)_2 \times 4\ H_2O$	134
**P13-Zn**	**P13**	2353	$Zn(OAc)_2 \times 2\ H_2O$	125
**P13-Co**		1742	$Co(OAc)_2 \times 4\ H_2O$	105

Table S12. Results of the elemental analyses and the DSC and TGA investigations for the metallopolymer networks containing BMA (**MP21** to **P26**).

Polymer	Found in elemental analysis [%]			T_g [°C]		T_d [°C]
	C	H	N	Range	Middle	
**P11-Zn**	66.05	9.50	1.56	37 to 55	46	270
**P11-Co**	65.70	9.33	1.57	38 to 58	48	268
**P12-Zn**	65.19	9.29	2.68	30 to 59	45	286
**P12-Co**	64.22	9.18	2.54	32 to 80	56	279
**P13-Zn**	64.17	9.05	2.46	33 to 64	49	276
**P13-Co**	64.34	9.10	2.50	39 to 67	54	272

Differential Scanning Calorimetry of the Polymer Networks (P1 to P13) and the Metallopolymer Networks (P1-Zn/Co to P13-Zn/Co)

Differential scanning calorimetry (DSC) was measured on a Netzsch DSC 204 F1 Phoenix instrument under a nitrogen atmosphere with a heating rate of 20 K min^{-1}.

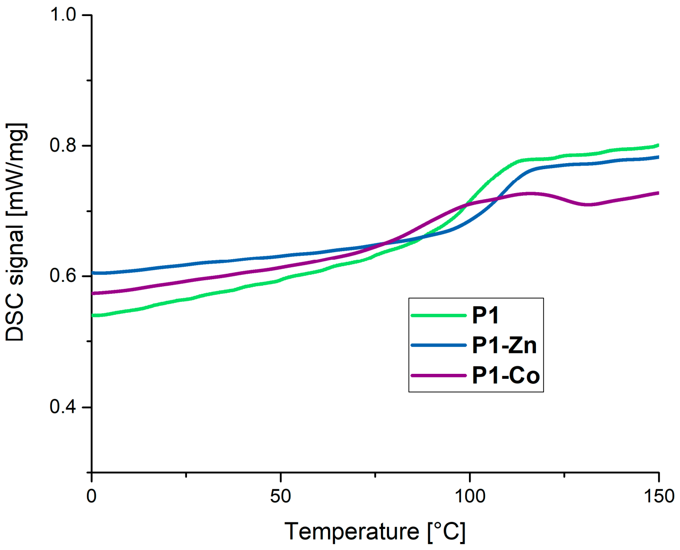

Figure S7. DSC curves of the polymer network **P1** (green) and the corresponding metallopolymer networks **P1-Zn** (blue) and **P1-Co** (purple).

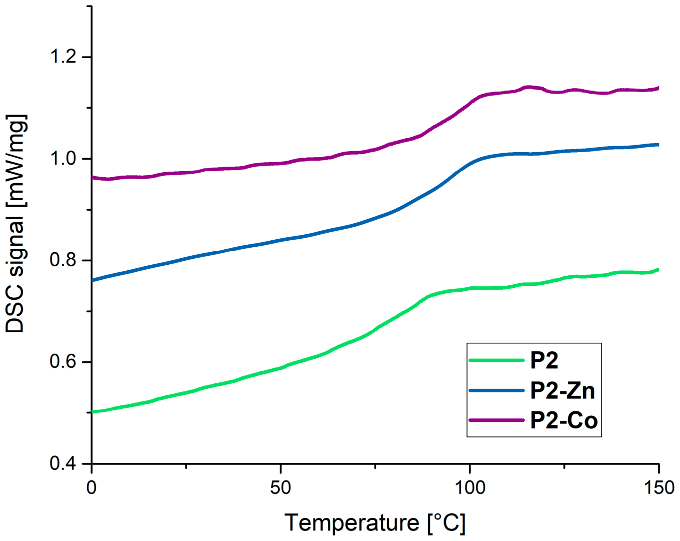

Figure S8. DSC curves of the polymer network **P2** (green) and the corresponding metallopolymer networks **P2-Zn** (blue) and **P2-Co** (purple).

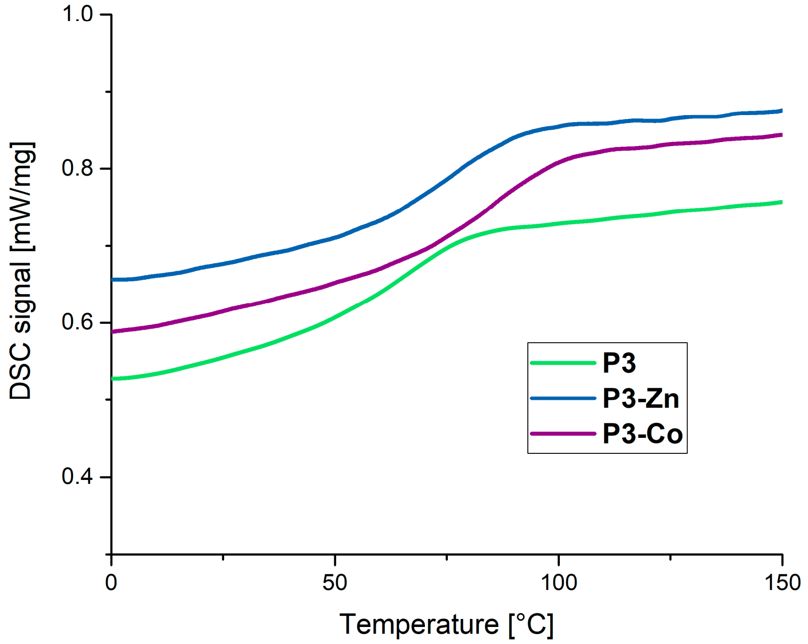

Figure S9. DSC curves of the polymer network **P3** (green) and the corresponding metallopolymer networks **P3-Zn** (blue) and **P3-Co** (purple).

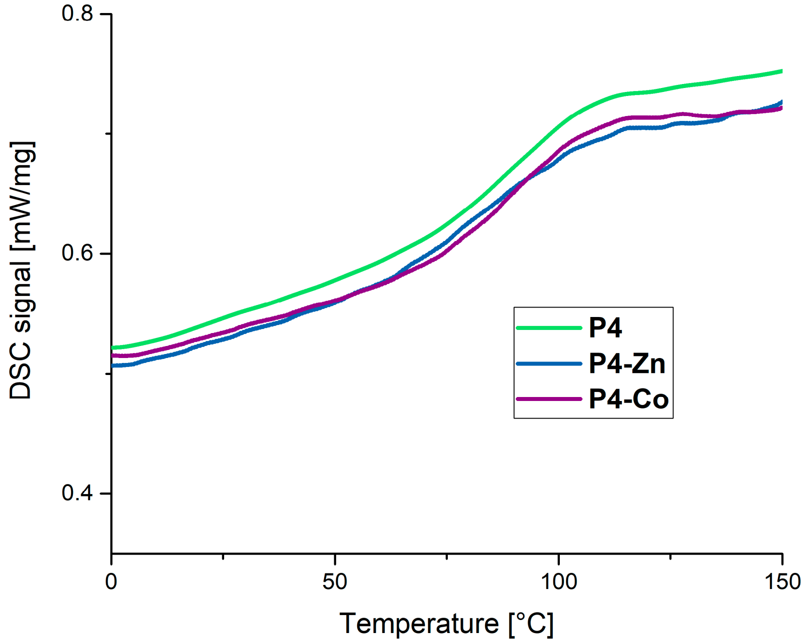

Figure S10. DSC curves of the polymer network **P4** (green) and the corresponding metallopolymer networks **P4-Zn** (blue) and **P4-Co** (purple).

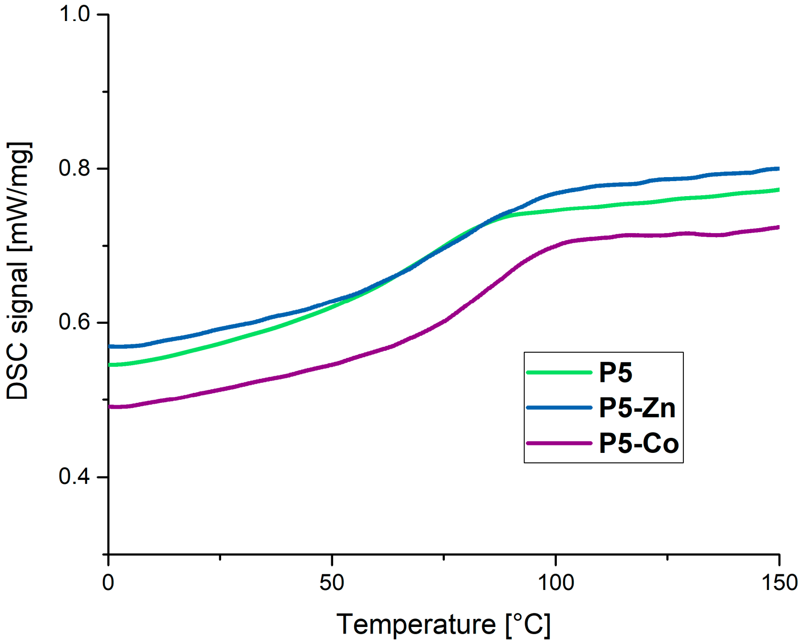

Figure S11. DSC curves of the polymer network **P5** (green) and the corresponding metallopolymer networks **P5-Zn** (blue) and **P5-Co** (purple).

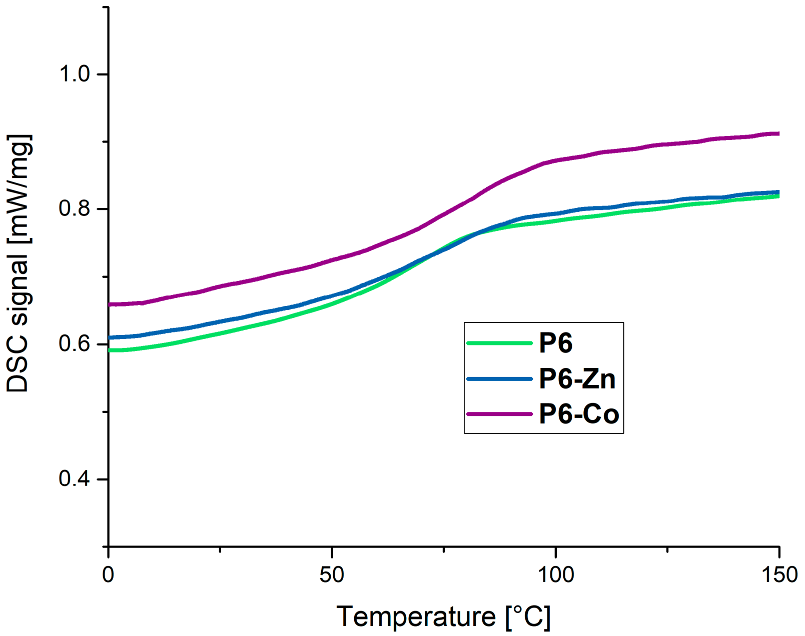

Figure S12. DSC curves of the polymer network **P6** (green) and the corresponding metallopolymer networks **P6-Zn** (blue) and **P6-Co** (purple).

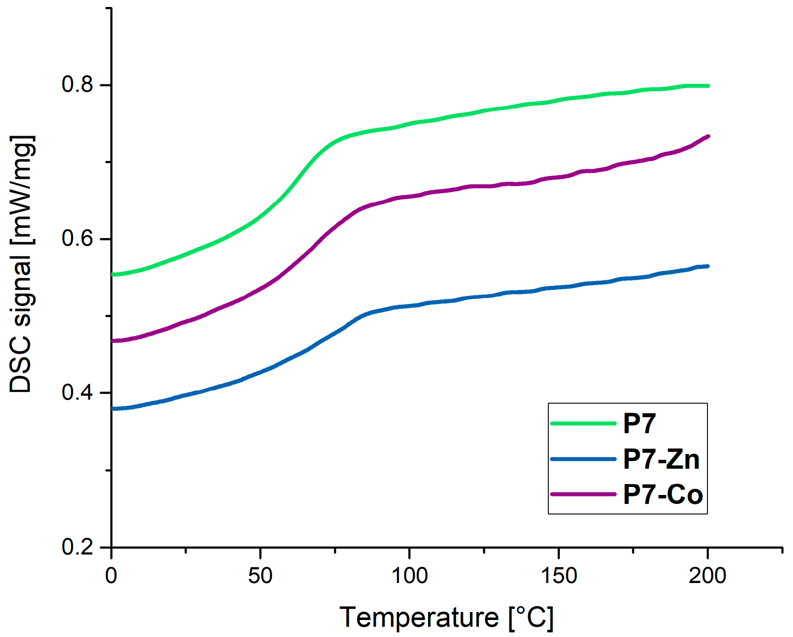

Figure S13. DSC curves of the polymer network **P7** (green) and the corresponding metallopolymer networks **P7-Zn** (blue) and **P7-Co** (purple).

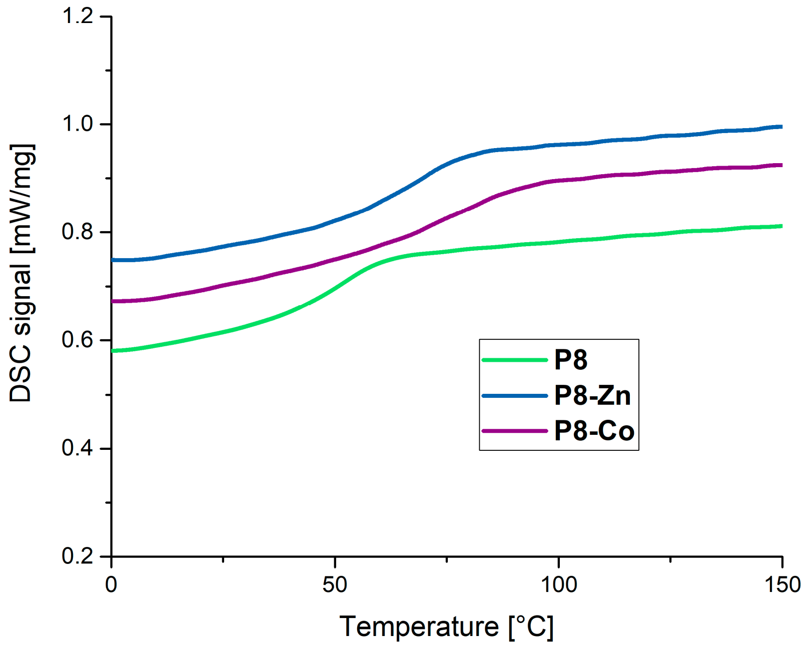

Figure S14. DSC curves of the polymer network **P8** (green) and the corresponding metallopolymer networks **P8-Zn** (blue) and **P8-Co** (purple).

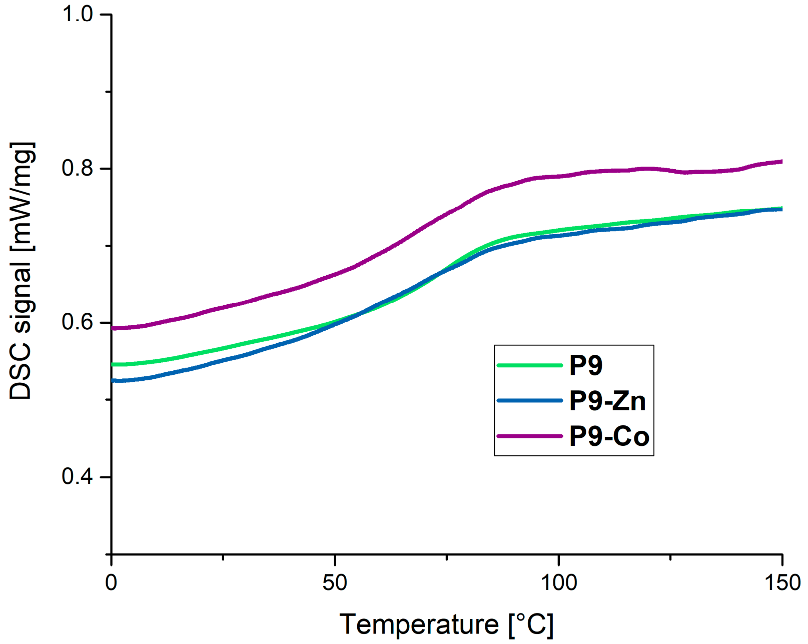

Figure S15. DSC curves of the polymer network **P9** (green) and the corresponding metallopolymer networks **P9-Zn** (blue) and **P9-Co** (purple).

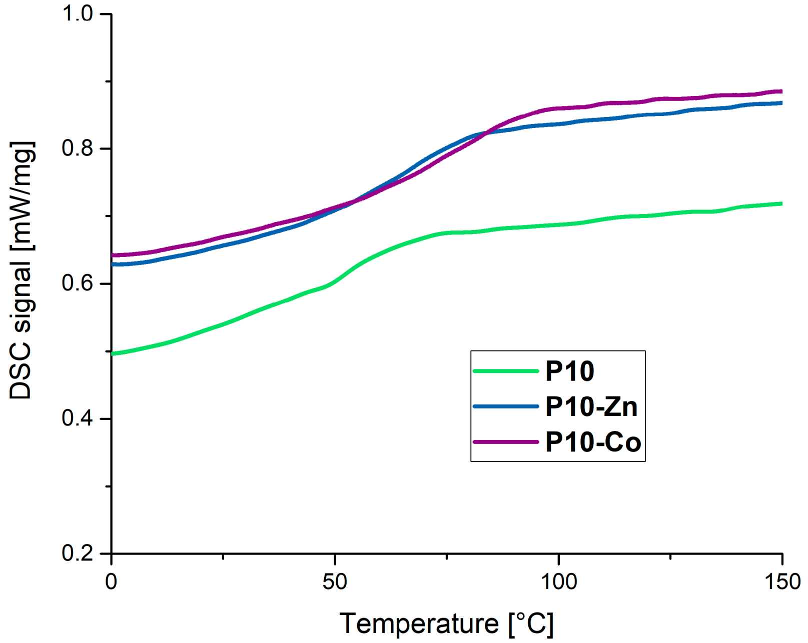

Figure S16. DSC curves of the polymer network **P10** (green) and the corresponding metallopolymer networks **P10-Zn** (blue) and **P10-Co** (purple).

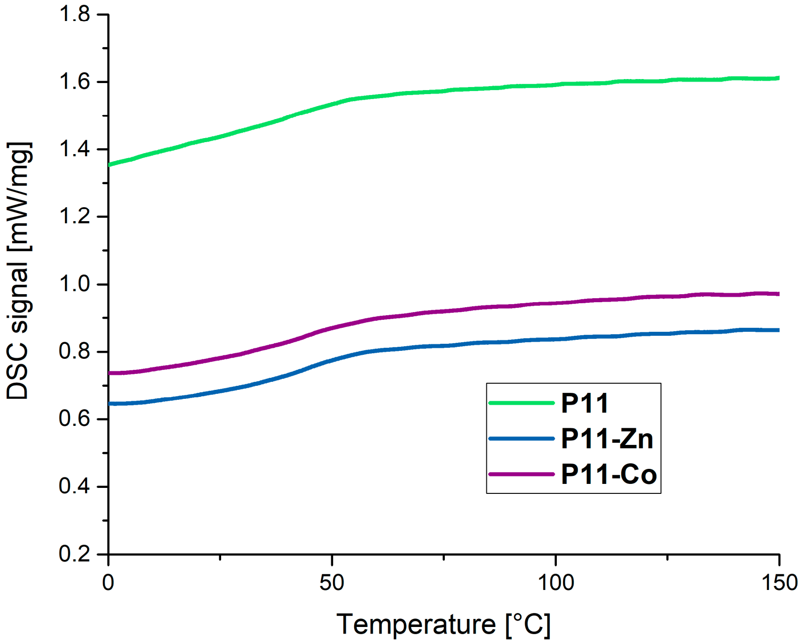

Figure S17. DSC curves of the polymer network **P11** (green) and the corresponding metallopolymer networks **P11-Zn** (blue) and **P11-Co** (purple).

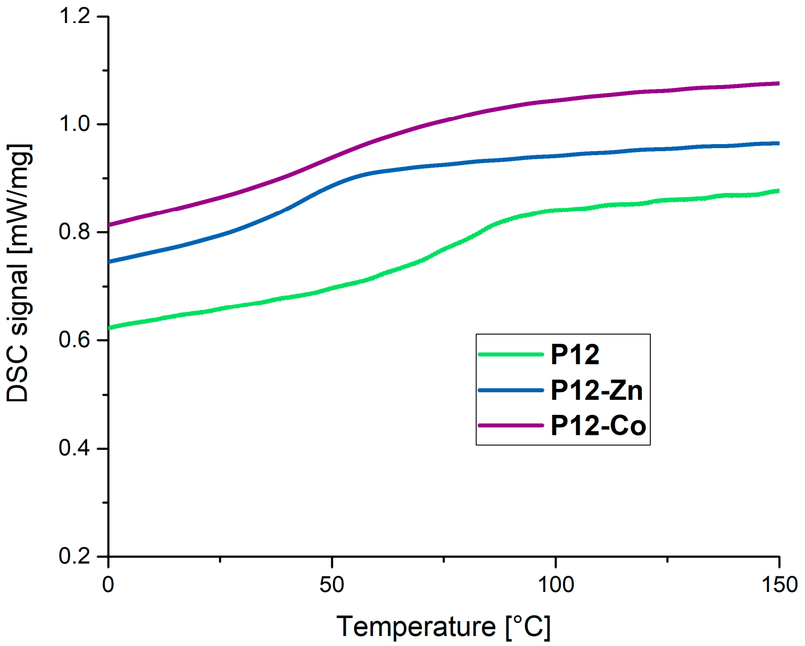

Figure S18. DSC curves of the polymer network **P12** (green) and the corresponding metallopolymer networks **P12-Zn** (blue) and **P12-Co** (purple).

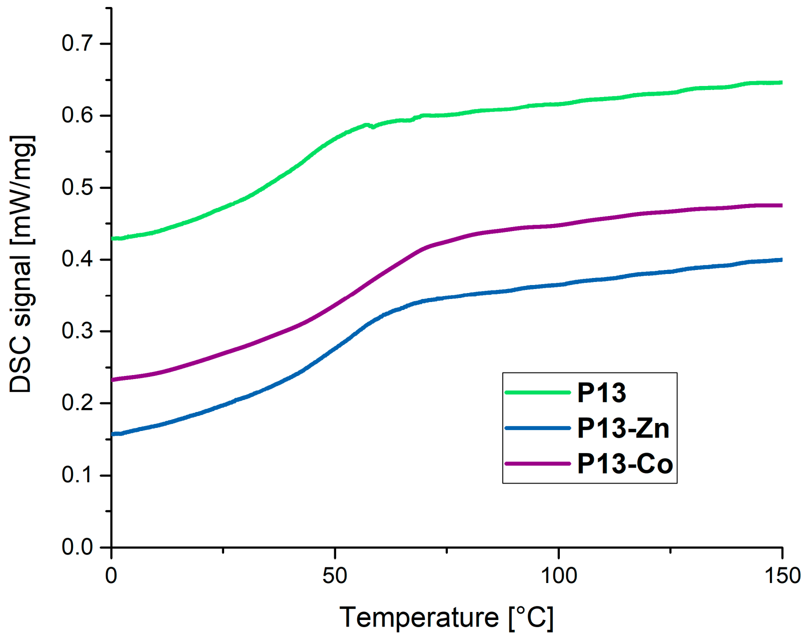

Figure S19. DSC curves of the polymer network **P13** (green) and the corresponding metallopolymer networks **P13-Zn** (blue) and **P13-Co** (purple).

Thermogravimetric Analysis (TGA) of the Polymer Networks (P1 to P13) and the Metallo-Polymer Networks (P1-Zn/Co to P13-Zn/Co)

The thermogravimetric analysis was carried under normal atmosphere using a Netzsch TG 209 F1.

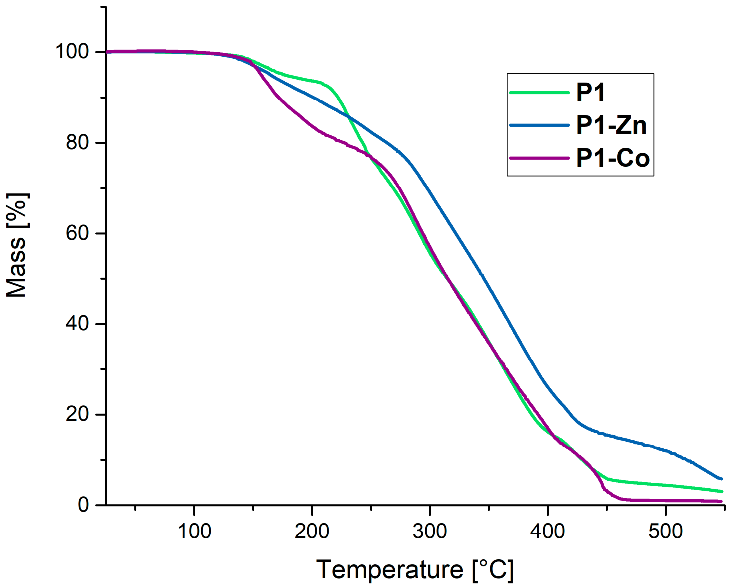

Figure S20. TGA curves of the polymer network **P1** (green) and the corresponding metallopolymer networks **P1-Zn** (blue) and **P1-Co** (purple).

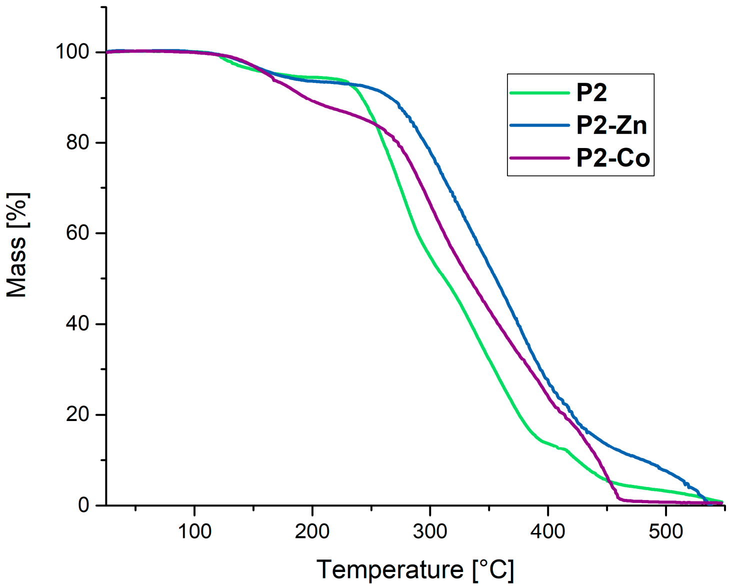

Figure S21. TGA curves of the polymer network **P2** (green) and the corresponding metallopolymer networks **P2-Zn** (blue) and **P2-Co** (purple).

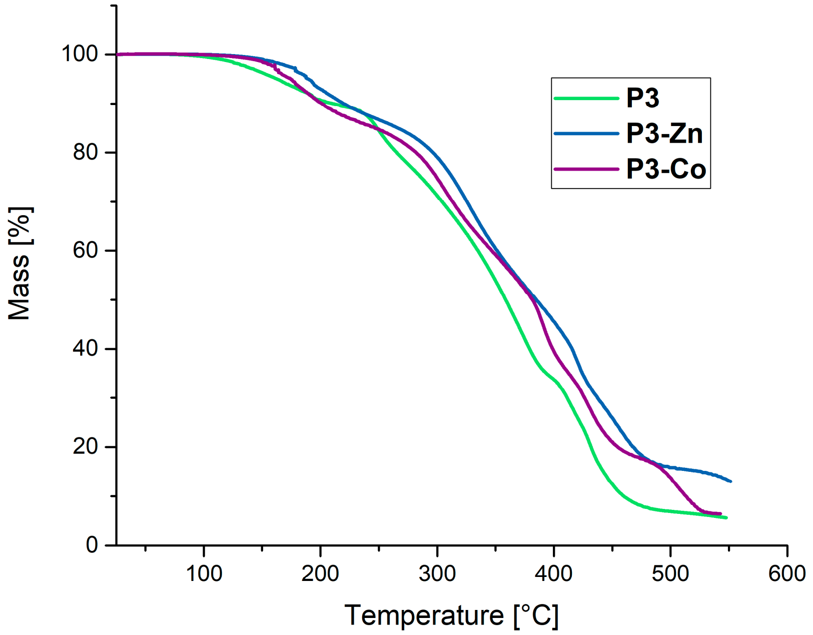

Figure S22. TGA curves of the polymer network **P3** (green) and the corresponding metallopolymer networks **P3-Zn** (blue) and **P3-Co** (purple).

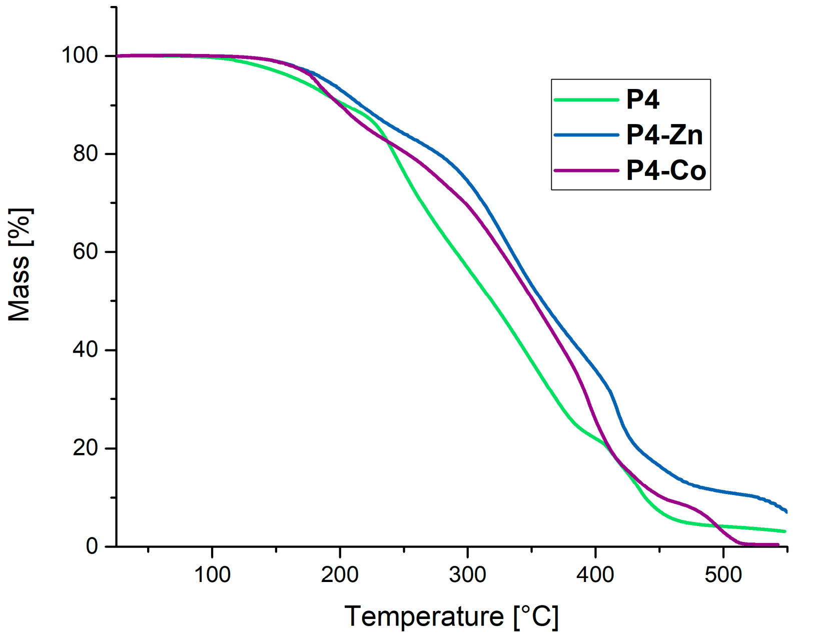

Figure S23. TGA curves of the polymer network **P4** (green) and the corresponding metallopolymer networks **P4-Zn** (blue) and **P4-Co** (purple).

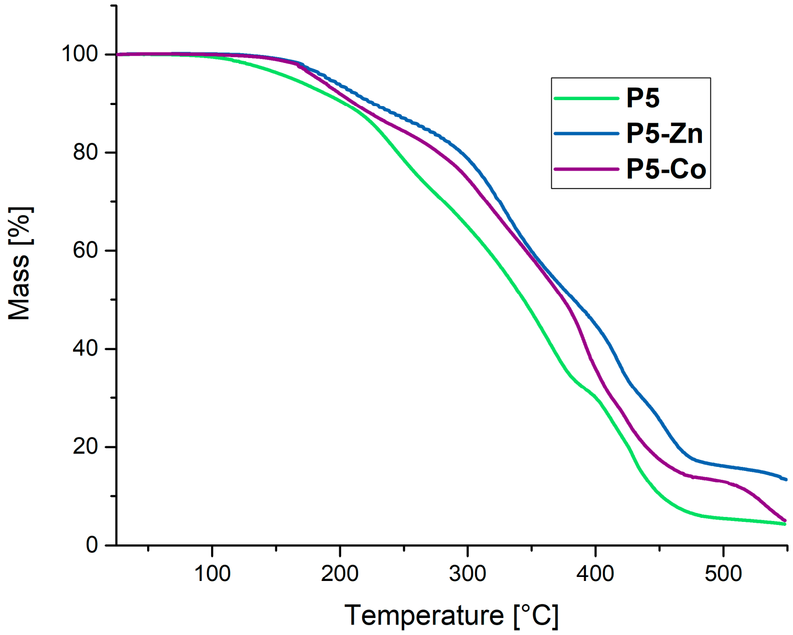

Figure S24. TGA curves of the polymer network **P5** (green) and the corresponding metallopolymer networks **P5-Zn** (blue) and **P5-Co** (purple).

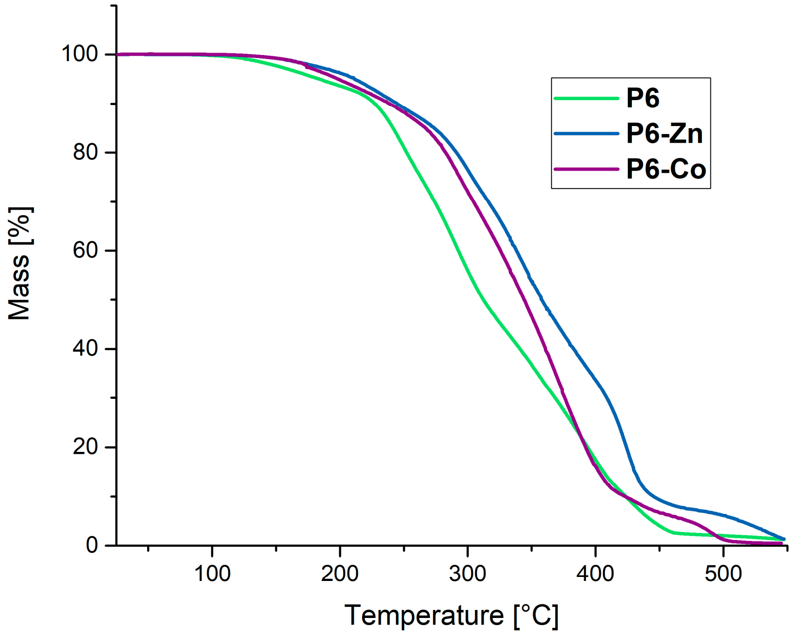

Figure S25. TGA curves of the polymer network **P6** (green) and the corresponding metallopolymer networks **P6-Zn** (blue) and **P6-Co** (purple).

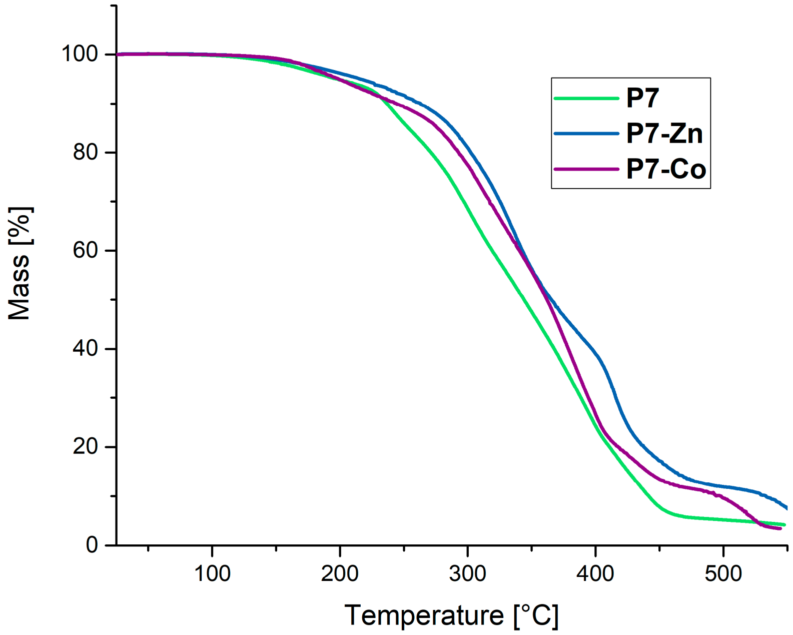

Figure S26. TGA curves of the polymer network **P7** (green) and the corresponding metallopolymer networks **P7-Zn** (blue) and **P7-Co** (purple).

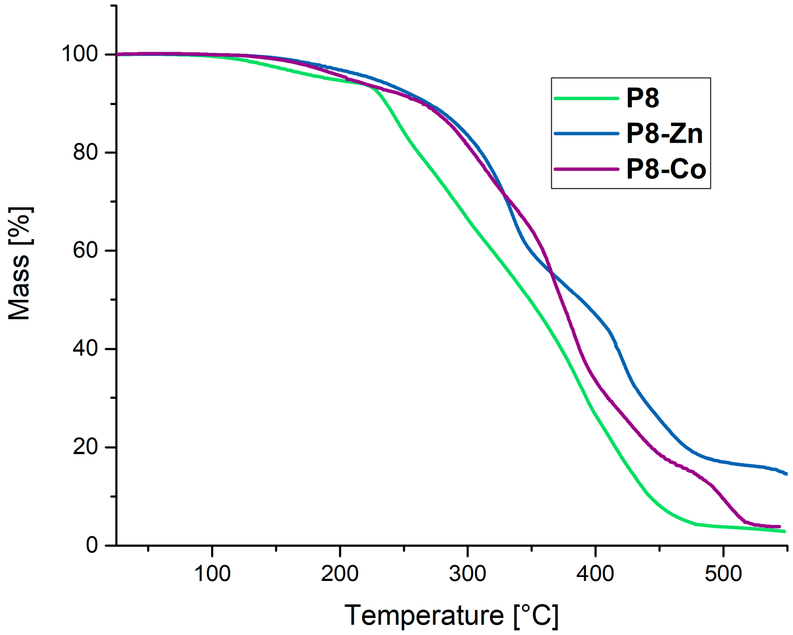

Figure S27. TGA curves of the polymer network **P8** (green) and the corresponding metallopolymer networks **P8-Zn** (blue) and **P8-Co** (purple).

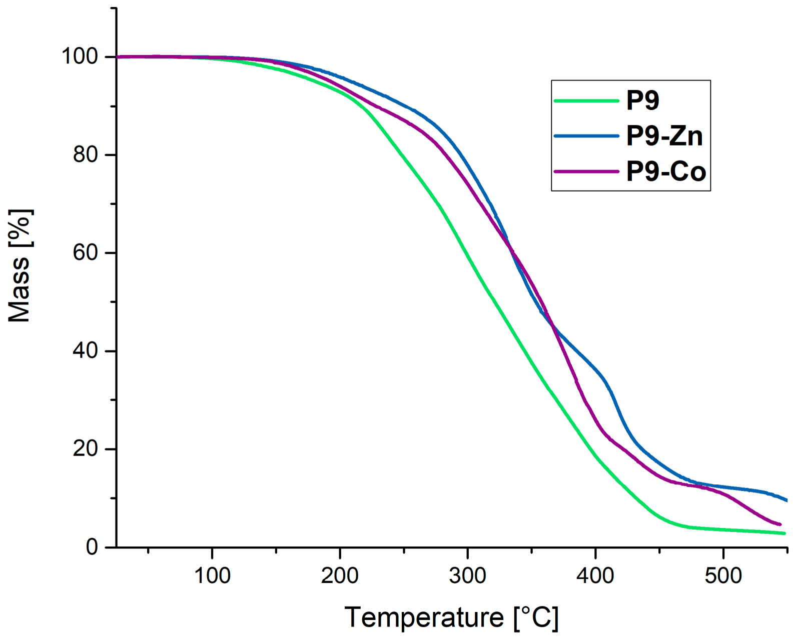

Figure S28. TGA curves of the polymer network **P9** (green) and the corresponding metallopolymer networks **P9-Zn** (blue) and **P9-Co** (purple).

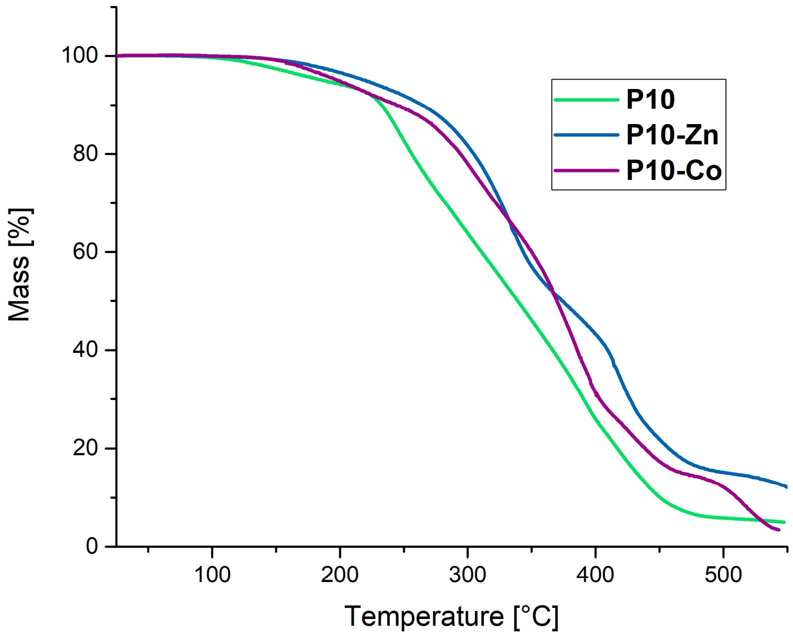

Figure S29. TGA curves of the polymer network **P10** (green) and the corresponding metallopolymer networks **P10-Zn** (blue) and **P10-Co** (purple).

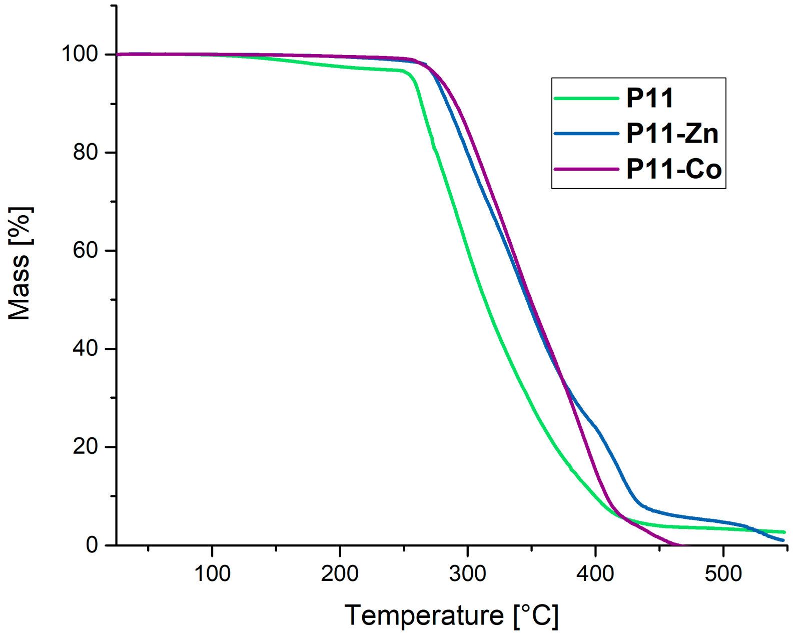

Figure S30. TGA curves of the polymer network **P11** (green) and the corresponding metallopolymer networks **P11-Zn** (blue) and **P11-Co** (purple).

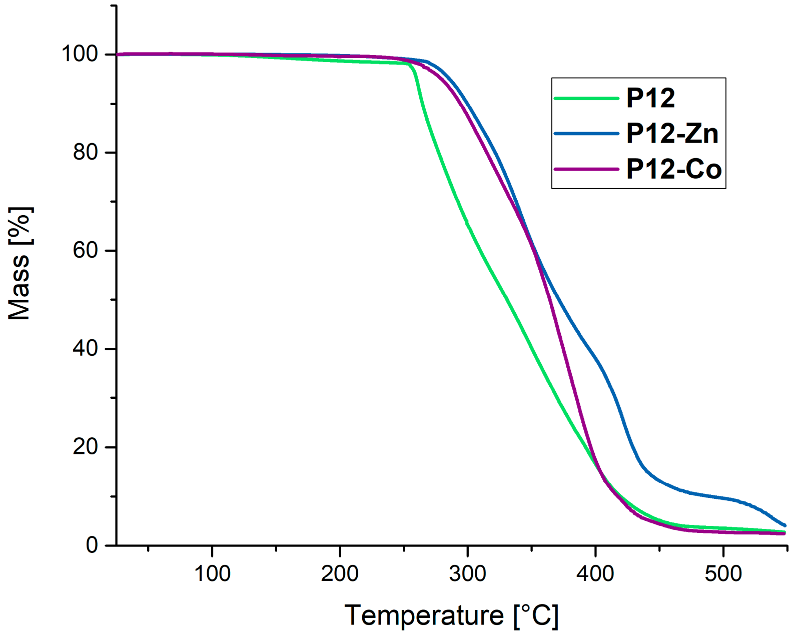

Figure S31. TGA curves of the polymer network **P12** (green) and the corresponding metallopolymer networks **P12-Zn** (blue) and **P12-Co** (purple).

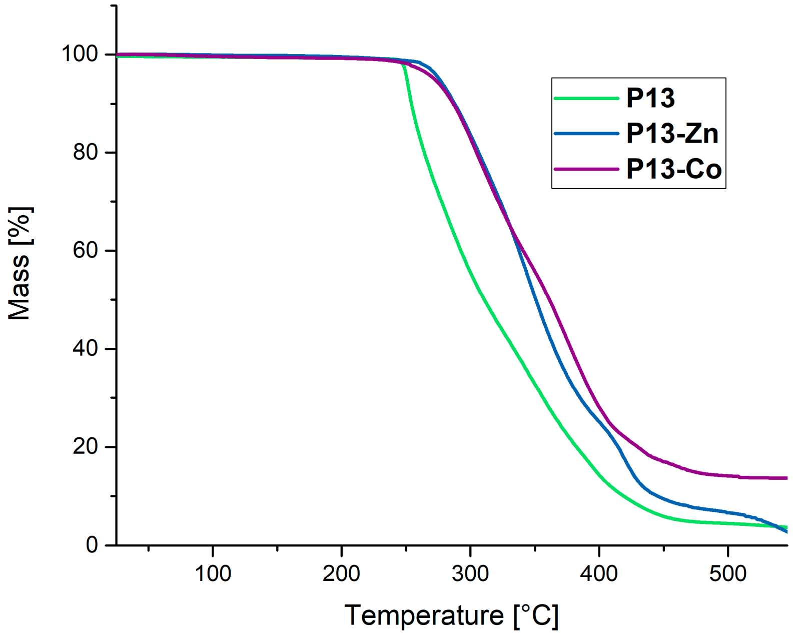

Figure S32. TGA curves of the polymer network **P13** (green) and the corresponding metallopolymer networks **P13-Zn** (blue) and **P13-Co** (purple).

NMR Spectra of the Polymer Networks (P1 to P13)

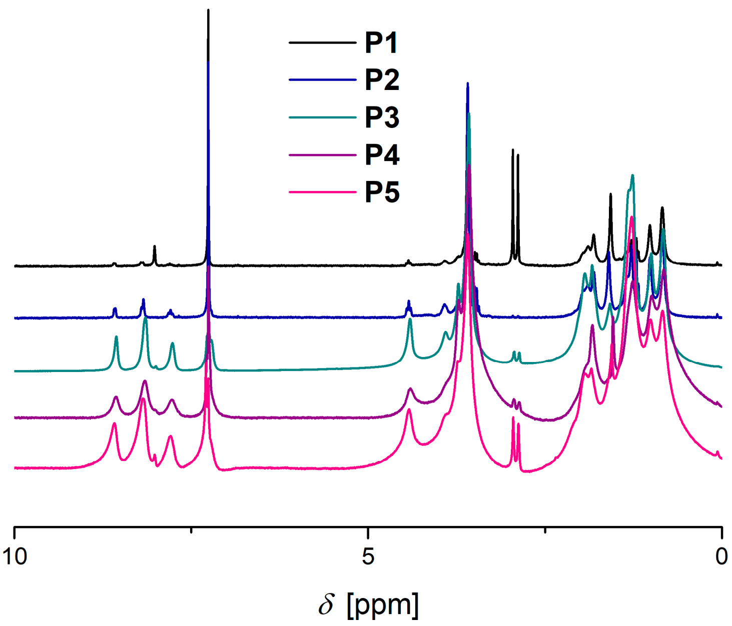

Figure S33. ^{1}H NMR spectrum of **P1** to **P5** (250 MHz, $CDCl_3$).

P1: ^{1}H NMR (250 MHz, $CDCl_3$, δ) : 0.63 – 2.07 (m, 149H, polymer-backbone, C*H*$_2$-alkyl chains), 3.32 – 4.13 (m, 67H, O-C*H*$_2$, O-C*H*$_3$), 4.43 (s, 2H, N-C*H*$_2$), 7.73 (s, 1H, pyridine-*H*), 8.21 (s, 2H, pyridine-*H*, triazole-*H*), 8.56 (s, 1H, pyridine-*H*) ppm.

P2: ^{1}H NMR (250 MHz, $CDCl_3$, δ) = 0.72 – 2.09 (m, 178H, polymer-backbone, C*H*$_2$-alkyl chains), 3.45 – 4.18 (m, 75H, O-C*H*$_2$, O-C*H*$_3$), 4.43 (s, 4H, N-C*H*$_2$), 7.76 (s, 2H, pyridine-*H*), 8.20 (s, 4H, pyridine-*H*, triazole-*H*), 8.58 (s, 2H, pyridine-*H*) ppm.

P3: ^{1}H NMR (400 MHz, $CDCl_3$, δ) = 0.16 – 2.46 (m, 189H, polymer-backbone, C*H*$_2$-alkyl chains), 3.10 – 5.24 (m, 89H, O-C*H*$_2$, O-C*H*$_3$, N-C*H*$_2$), 7.22 (s, 4H, pyridine-*H*), 7.76 (s, 4H, pyridine-*H*), 8.15 (s, 8H, pyridine-*H*, triazole-*H*), 8.56 (s, 4H, pyridine-*H*) ppm..

P4: ^{1}H NMR (250 MHz, $CDCl_3$, δ) = 0.88 – 2.12 (m, 158H, polymer-backbone, C*H*$_2$-alkyl chains), 2.52 – 4.72 (m, 97H, O-C*H*$_2$, O-C*H*$_3$, N-C*H*$_2$), 7.78 (s, 2H, pyridine-*H*), 8.15 (s, 4H, pyridine-*H*, triazole-*H*), 8.56 (s, 2H, pyridine-*H*) ppm.

P5: : ^{1}H NMR (250 MHz, $CDCl_3$, δ) = 0.59– 2.36 (m, 161H, polymer-backbone, C*H*$_2$-alkyl chains), 3.02 – 4.98 (m, 76H, O-C*H*$_2$, O-C*H*$_3$, N-C*H*$_2$), 7.78 (s, 2H, pyridine-*H*), 8.19 (s, 4H, pyridine-*H*, triazole-*H*), 8.60 (s, 2H, pyridine-*H*) ppm.

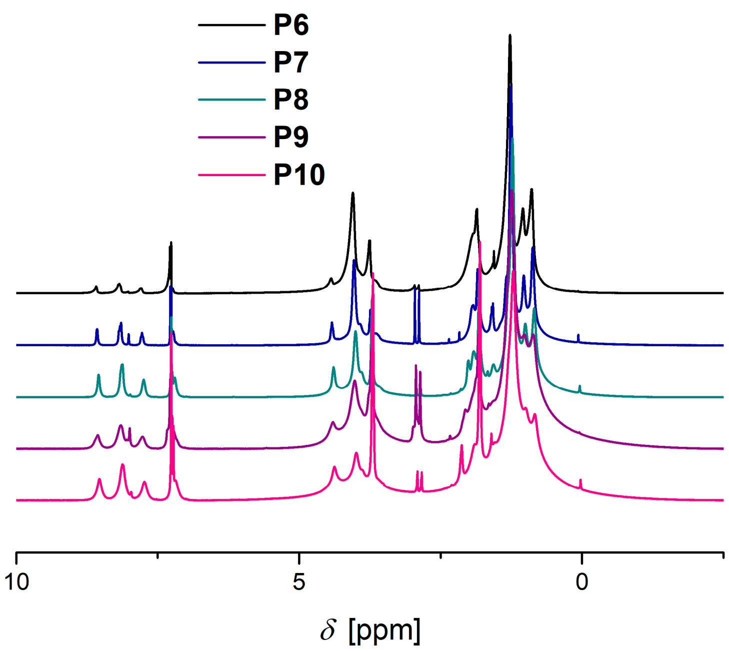

Figure S34. ^1H NMR spectrum of **P6** to **P10** (250 MHz, $CDCl_3$).

P6: ^1H NMR (400 MHz, $CDCl_3$, δ) = 0.59 – 2.30 (m, 185H, polymer-backbone, C*H*$_2$-alkyl chains, CH_2-C*H*$_3$), 3.52 – 4.63 (m, 56H, O-C*H*$_2$, N-C*H*$_2$), 7.81 (s, 1H, pyridine-*H*), 8.20 (s, 2H, pyridine-*H*, triazole-*H*), 8.59 (s, 1H, pyridine-*H*) ppm.

P7: ^1H NMR (400 MHz, $CDCl_3$, δ) = 0.48 – 2.14 (m, 195H, polymer-backbone, C*H*$_2$-alkyl chains, CH_2-C*H*$_3$), 3.42 – 4.64 (m, 52H, O-C*H*$_2$, N-C*H*$_2$), 7.23 (s, 2H, pyridine-*H*), 7.78 (s, 2H, pyridine-*H*), 8.17 (s, 4H, pyridine-*H*, triazole-*H*), 8.57 (s, 2H, pyridine-*H*) ppm.

P8: ^1H NMR (400 MHz, $CDCl_3$, δ) = 0.14 – 2.28 (m, 266H, polymer-backbone, C*H*$_2$-alkyl chains, CH_2-C*H*$_3$), 3.27 – 4.80 (m, 70H, O-C*H*$_2$, N-C*H*$_2$), 7.20 (s, 4H, pyridine-*H*), 7.75 (s, 4H, pyridine-*H*), 8.13 (s, 8H, pyridine-*H*, triazole-*H*), 8.54 (s, 4H, pyridine-*H*) ppm; yield.

P9: ^1H NMR (400 MHz, $CDCl_3$, δ) = 0.40 – 2.16 (m, 223H, polymer-backbone, C*H*$_2$-alkyl chains, CH_2-C*H*$_3$), 3.12 – 4.92 (m, 68H, O-C*H*$_2$, N-C*H*$_2$), 7.22 (s, 2H, pyridine-*H*), 7.76 (s, 2H, pyridine-*H*), 8.15 (s, 4H, pyridine-*H*, triazole-*H*), 8.56 (s, 2H, pyridine-*H*) ppm.

P10: ^1H NMR (400 MHz, $CDCl_3$, δ) = 0.33 – 2.16 (m, 263H, polymer-backbone, C*H*$_2$-alkyl chains, CH_2-C*H*$_3$), 3.22 – 4.98 (m, 81H, O-C*H*$_2$, N-C*H*$_2$), 7.19 (s, 4H, pyridine-*H*), 7.73 (s, 4H, pyridine-*H*), 8.12 (s, 8H, pyridine-*H*, triazole-*H*), 8.53 (s, 4H, pyridine-*H*) ppm.

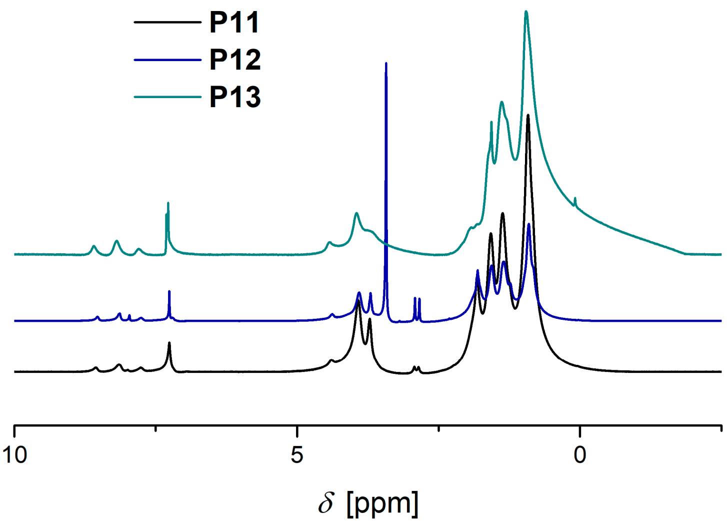

Figure S35. ^{1}H NMR spectrum of **P11** to **P13** (250 MHz, $CDCl_3$).

P11: ^{1}H NMR (300 MHz, $CDCl_3$; δ) = 0.62 – 1.94 (m, 335H, polymer-backbone, C*H*$_2$-alkyl chains, CH_2-C*H*$_3$), 3.38 – 4,56 (m, 50H, O-C*H*$_2$, N-C*H*$_2$), 7.77 (s, 1H, pyridine-*H*), 8.16 (s, 2H, pyridine-*H*, triazole-*H*), 8.58 (s, 1H, pyridine-*H*) ppm.

P12: ^{1}H NMR (600 MHz, $CDCl_3$, δ) = 0.60 – 1.91 (m, 365H, polymer-backbone, C*H*$_2$-alkyl chains, CH_2-C*H*$_3$), 3.18 – 4.96 (m, 48H, O-C*H*$_2$, N-C*H*$_2$), 7.23 (s, 2H, pyridine-*H*), 7.78 (s, 2H, pyridine-*H*), 8.16 (s, 4H, pyridine-*H*, triazole-*H*), 8.57 (s, 4H, pyridine-*H*) ppm.

P13: ^{1}H NMR (250 MHz, $CDCl_3$, δ) = 0.42 – 1.89 (m, 424H, polymer-backbone, C*H*$_2$-alkyl chains, CH_2-C*H*$_3$), 2.65 – 4.99 (m, 59H, O-C*H*$_2$, N-C*H*$_2$), 7.21 (s, 2H, pyridine-*H*), 7.80 (s, 2H, pyridine-*H*), 8.19 (s, 4H, pyridine-*H*, triazole-*H*), 8.59 (s, 4H, pyridine-*H*) ppm.

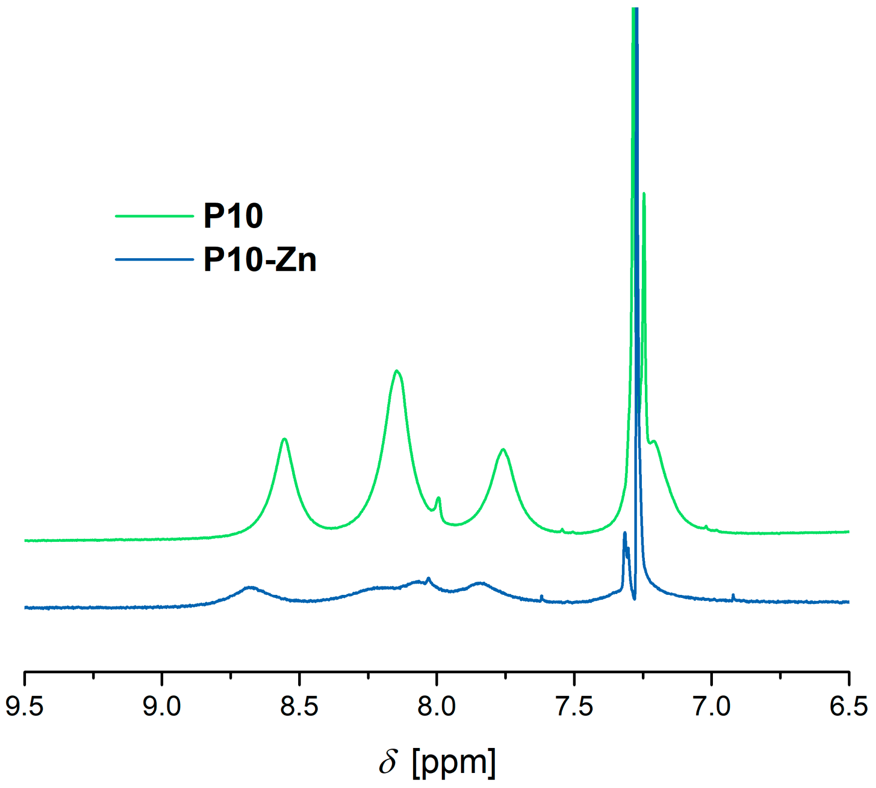

Figure S36. Zoom of the ^{1}H NMR spectrum of **P10** and **P10-Zn** (250 MHz, $CDCl_3$).

IR Spectroscopic Investigation of the Polymer (P1 to P13) and Metallo-polymer Networks (P1-Zn/Co to P13-Zn/Co)

FT-IR spectra were recorded from 600 up to 4000 cm^{-1} using an IR-Affinity 1.

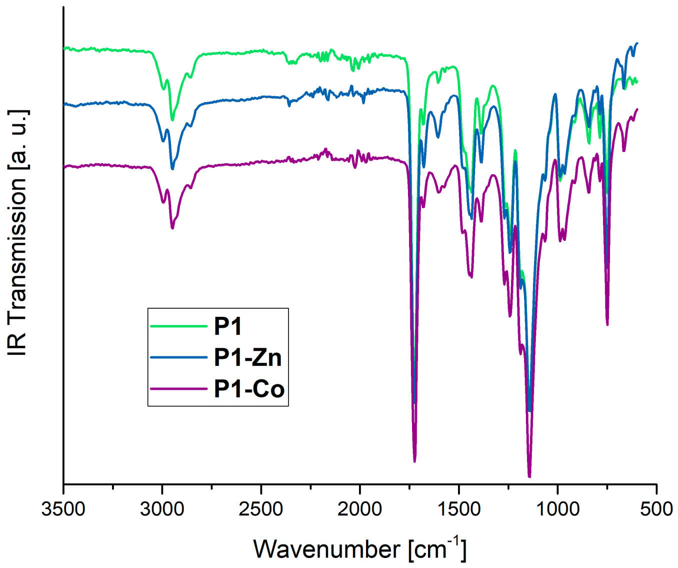

Figure S37. IR spectra of the polymer network **P1** (green) and the corresponding metallopolymer networks **P1-Zn** (blue) and **P1-Co** (purple).

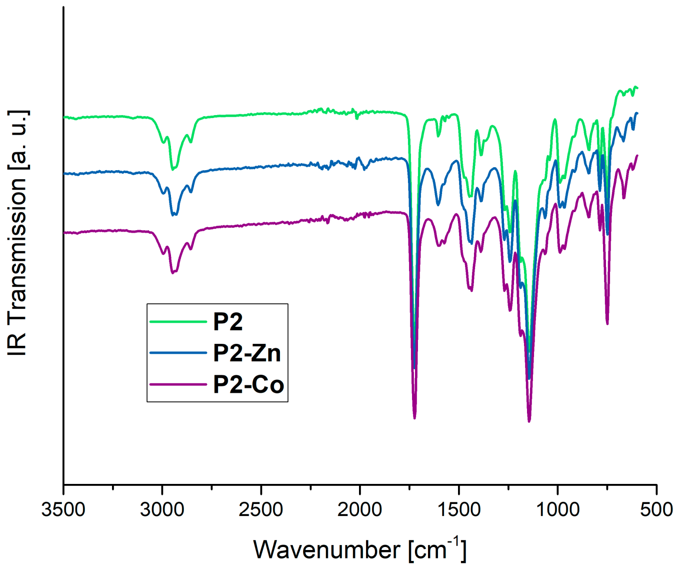

Figure S38. IR spectra of the polymer network **P2** (green) and the corresponding metallopolymer networks **P2-Zn** (blue) and **P2-Co** (purple).

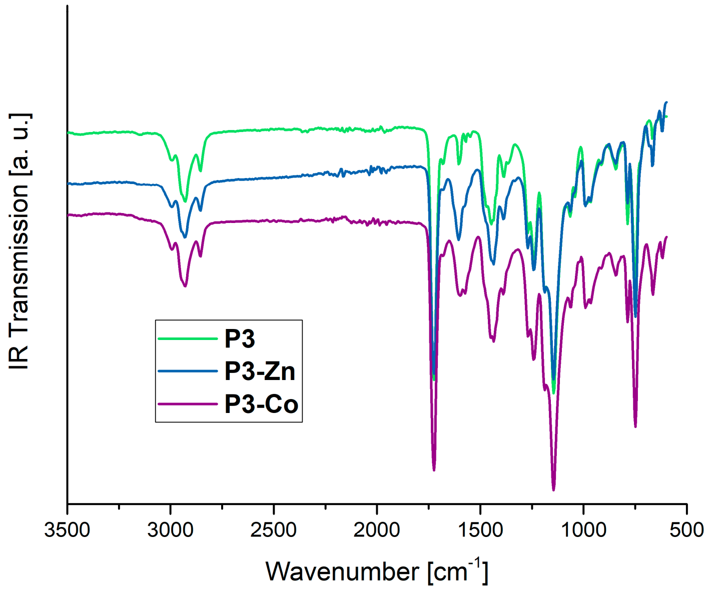

Figure S39. IR spectra of the polymer network **P3** (green) and the corresponding metallopolymer networks **P3-Zn** (blue) and **P3-Co** (purple).

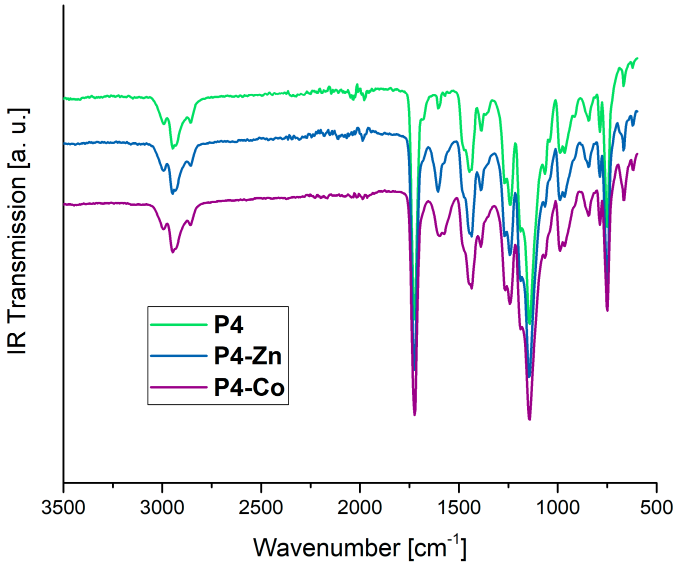

Figure S40. IR spectra of the polymer network **P4** (green) and the corresponding metallopolymer networks **P4-Zn** (blue) and **P4-Co** (purple).

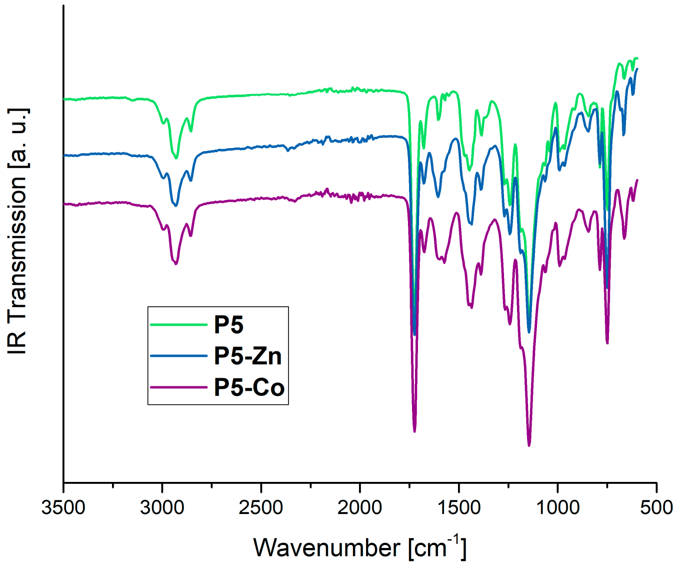

Figure S41. IR spectra of the polymer network **P5** (green) and the corresponding metallopolymer networks **P5-Zn** (blue) and **P5-Co** (purple).

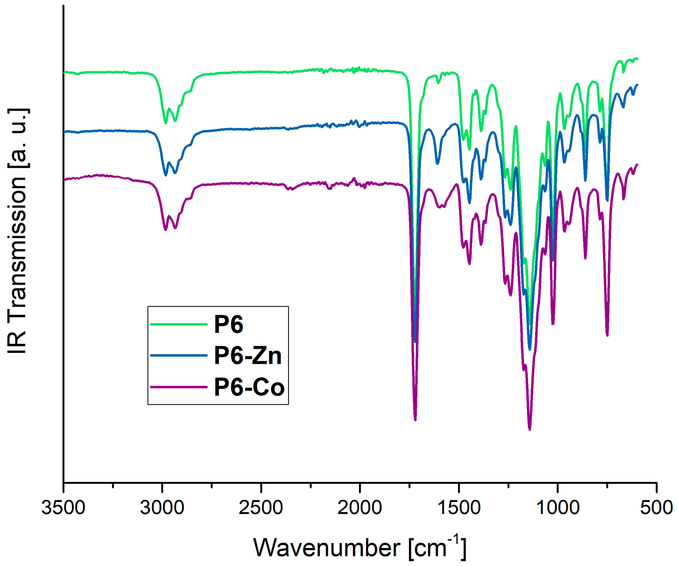

Figure S42. IR spectra of the polymer network **P6** (green) and the corresponding metallopolymer networks **P6-Zn** (blue) and **P6-Co** (purple).

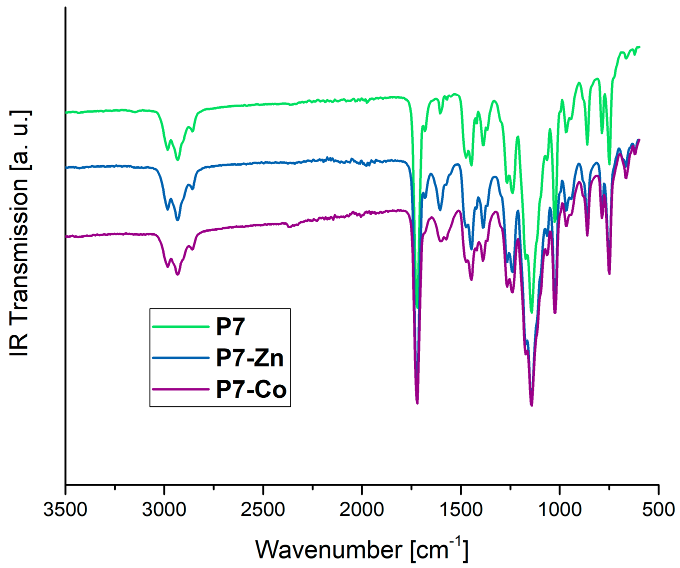

Figure S43. IR spectra of the polymer network **P7** (green) and the corresponding metallopolymer networks **P7-Zn** (blue) and **P7-Co** (purple).

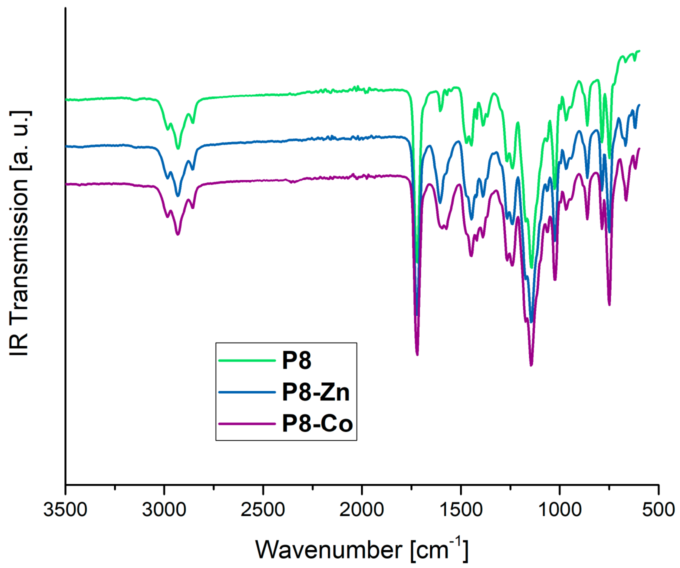

Figure S44. IR spectra of the polymer network **P8** (green) and the corresponding metallopolymer networks **P8-Zn** (blue) and **P8-Co** (purple).

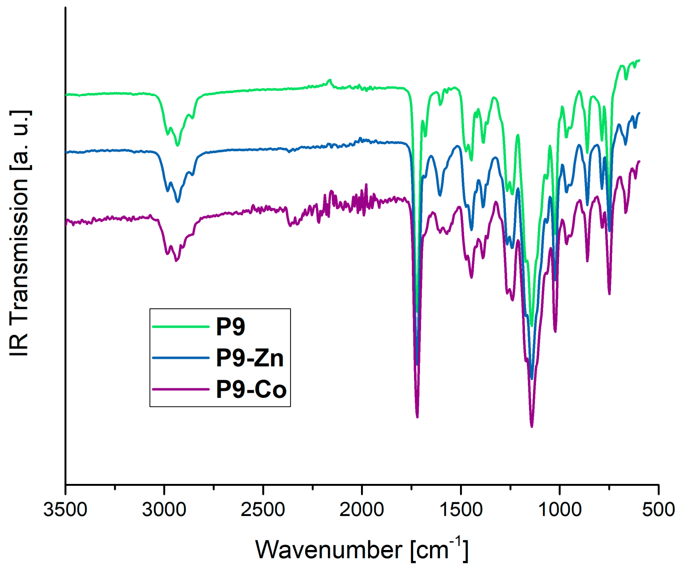

Figure S45. IR spectra of the polymer network P9 (green) and the corresponding metallopolymer networks P9-Zn (blue) and P9-Co (purple).

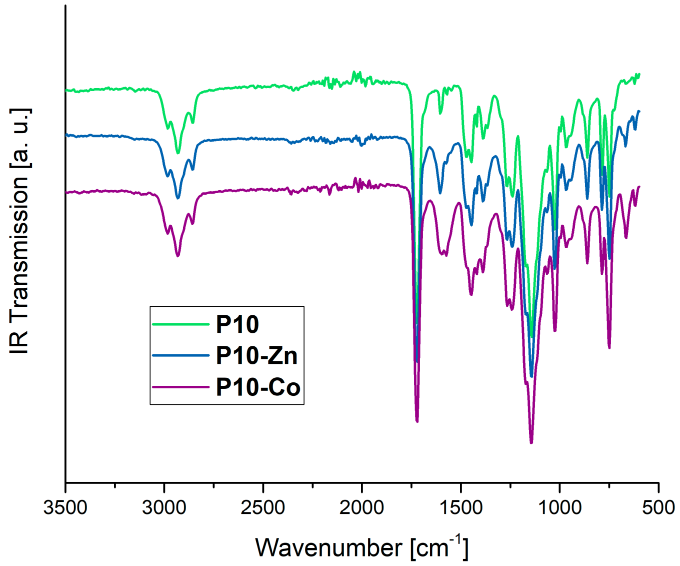

Figure S46. IR spectra of the polymer network **P10** (green) and the corresponding metallopolymer networks **P10-Zn** (blue) and **P10-Co** (purple).

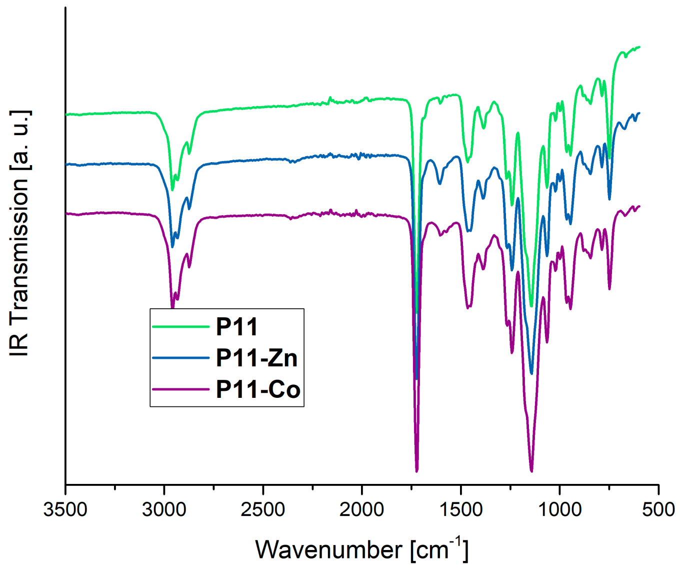

Figure S47. IR spectra of the polymer network **P11** (green) and the corresponding metallopolymer networks **P11-Zn** (blue) and **P11-Co** (purple).

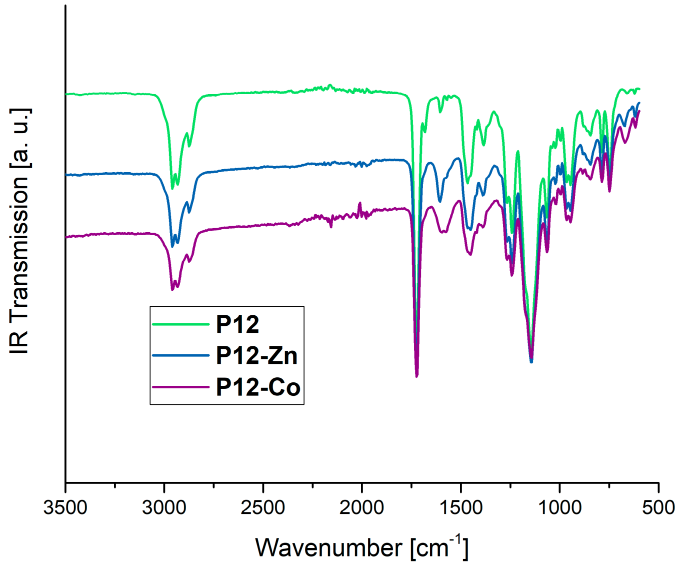

Figure S48. IR spectra of the polymer network **P1** (green) and the corresponding metallopolymer networks **P12-Zn** (blue) and **P12-Co** (purple).

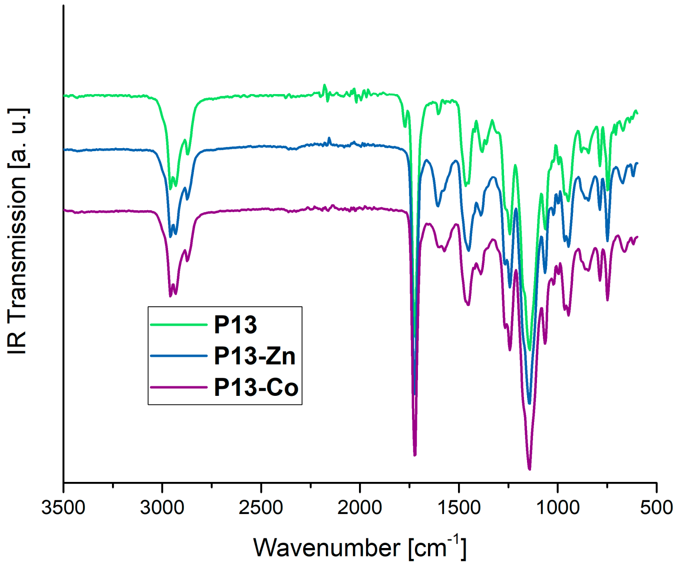

Figure S49. IR spectra of the polymer network **P13** (green) and the corresponding metallopolymer networks **P13-Zn** (blue) and **P13-Co** (purple).

P1 to P5
FT-IR (cm^{-1}): 664, 748, 841, 988, 1146, 1238, 1385, 1435, 1605, 1678, 1724, 2947.

P6 to P10
FT-IR (cm^{-1}): 663, 784, 968, 1026, 1142, 1238, 1389, 1447, 1474, 1605, 1721, 2936.

P11 to P13
FT-IR (cm^{-1}): 664, 748, 841, 964, 1065, 1142, 1242, 1269, 1466, 1605, 1725, 2932, 2959.

Temperature Dependent Raman Spectroscopy of P12-Zn

FT-Raman spectra were recorded up to 4000 cm^{-1} with a spectral resolution of 4 cm^{-1} using a commercial Bruker MultiSpec spectrometer. The Raman excitation light at 1064 nm was provided by a Nd:YAG laser (Klastech DeniCAFC-LC-3/40). The laser power at the samples was 1000 mW. The FT-Raman spectra were recorded using the software package OPUS 6.5. To analyze the temperature-dependent behavior of **P12-Zn** temperature-dependent Raman spectra were recorded. The samples were heated *via* a Linkam stage LTS 350 with a heating rate of 1 °C/min. Five Raman spectra were recorded at 27 °C before the heating to 150 °C was started. A Raman spectrum consisting of 32 single scans was recorded every minute during the heating process. The raw Raman spectra were pre-processed using R (3.5.1). First the Raman spectra were restricted to the wavenumber region of interest, *i.e.* the region between 400 and 3200 cm^{-1}. Subsequently, the Raman spectra were background corrected using a SNIP algorithm (iterations = 50, order = 2, smoothing window = 3) and normalized to the CH-stretching area (2800 to 3100 cm^{-1}).

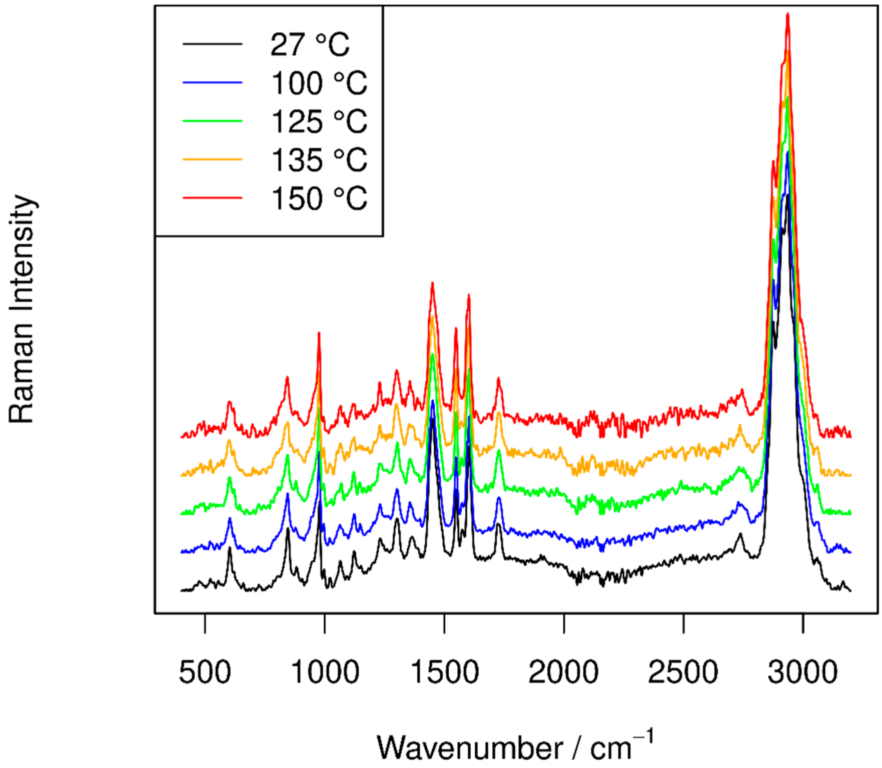

Figure S50. FT-Raman spectra of the metallopolymer network (**P12-Zn**) at different temperatures (Black: 27 °C; blue: 100 °C, green: 125 °C; orange: 135 °C; red: 150 °C).

Cyclo-mechanic-tests of Selected Metallopolymer Networks

Table S13. Results of the cyclo-mechanic-tests of the metallopolymer networks **P1-Zn, P6-Zn, P7-Zn, P8-Zn, P11-Zn**.

Metallo polymer	Cycle	ε_p (N-1) [%]	ε_m (N) [%]	ε_u (N) [%]	ε_p (N) [%]
**P1-Zn**	1	0	5.3	5.3	0.386
(MMA, 5% crosslinker,	2	0	3.9	3.9	0.75
5% ligand)	3	0	3.7	3.7	0.671
**P6-Zn**	1	0	31.2	30.8	1.52
(EMA, 5% crosslinker, 5%	2	0	30.6	30.2	1.53
ligand)	3	0	30.0	29.6	1.13
**P7-Zn**	1	0	13.2	13.0	0.638
(EMA, 5% crosslinker,	2	0	12.6	12.4	0.583
10% ligand)	3	0	12.2	12.0	0.565
**P8-Zn**	1	0	27.2	26.8	1.02
(EMA, 5% crosslinker,	2	0	25.3	24.8	0.633
20% ligand)	3	0	25.0	24.5	0.456
**P11-Zn**	1	0	11.4	11.3	0.386
(BMA, 5% crosslinker, 5%	2	0	10.6	10.5	0.450
ligand)	3	0	10.4	10.4	0.356

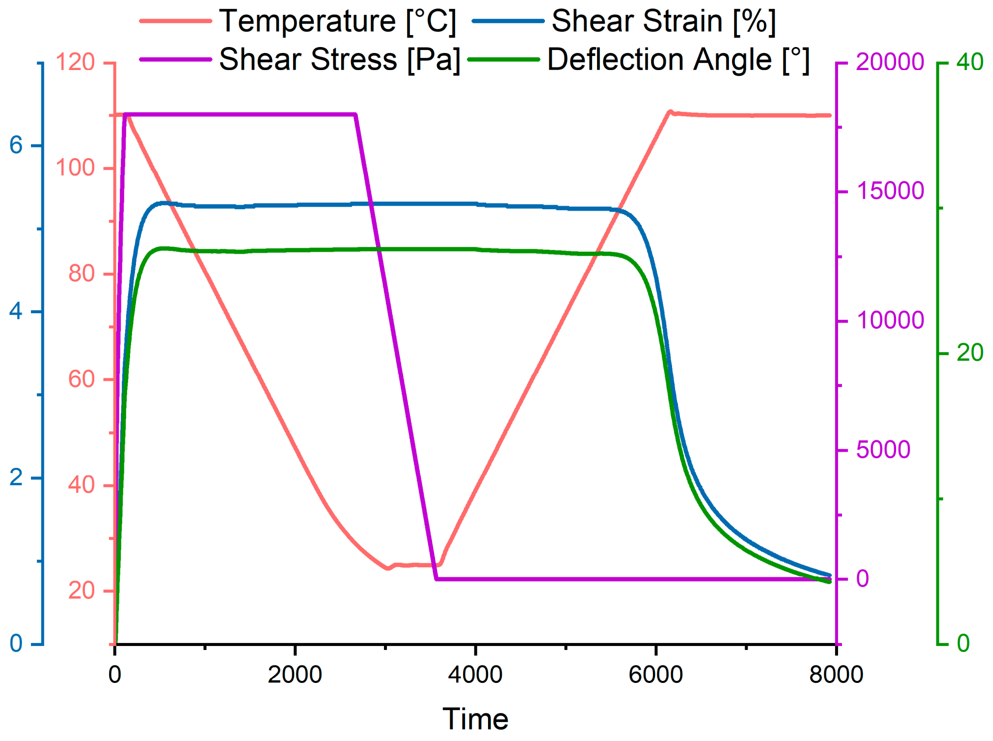

Figure S51. First cycle of the cyclo mechanic test of the metallopolymer networks **P1-Zn**.

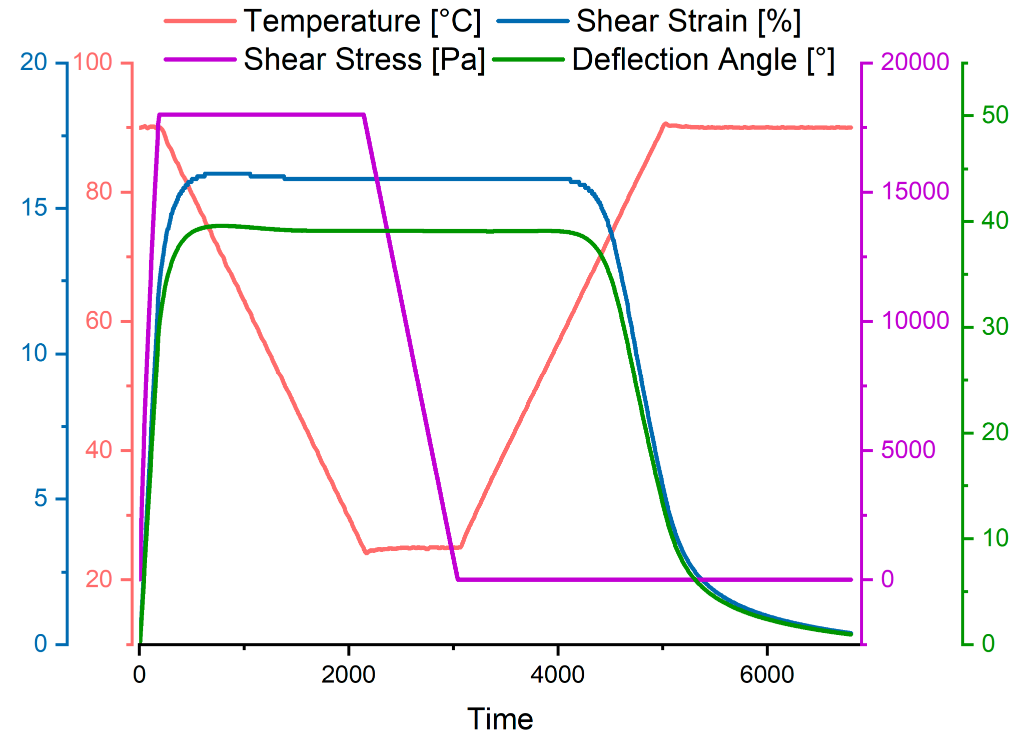

Figure S52. First cycle of the cyclo mechanic test of the metallopolymer networks **P6-Zn**.

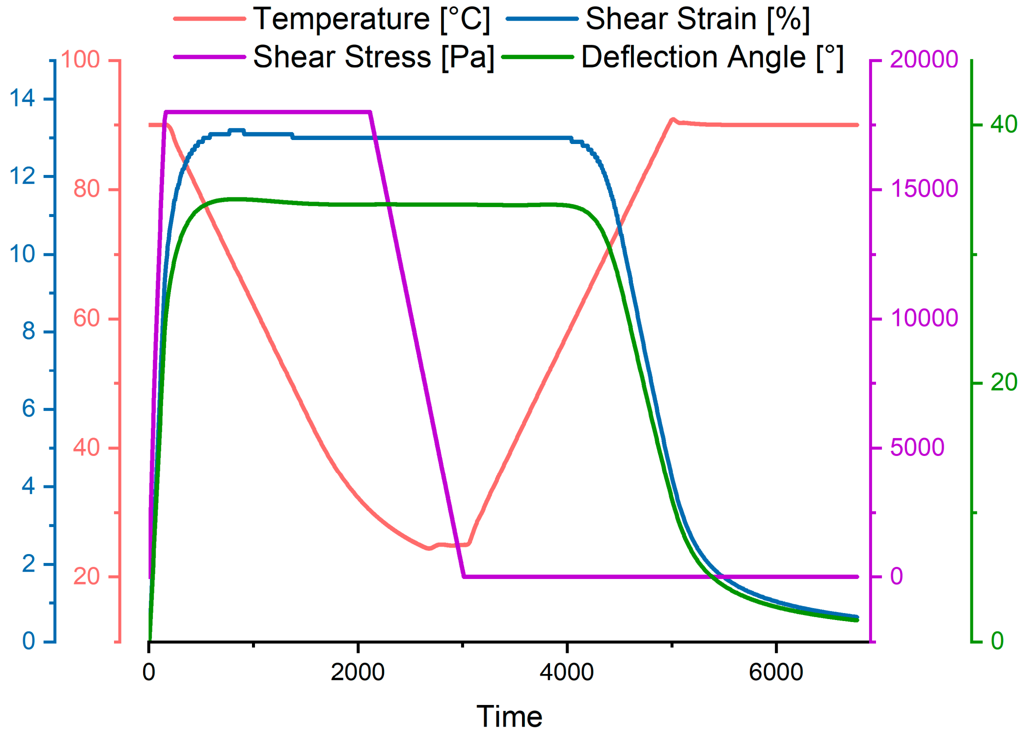

Figure S53. First cycle of the cyclo mechanic test of the metallopolymer networks **P7-Zn**.

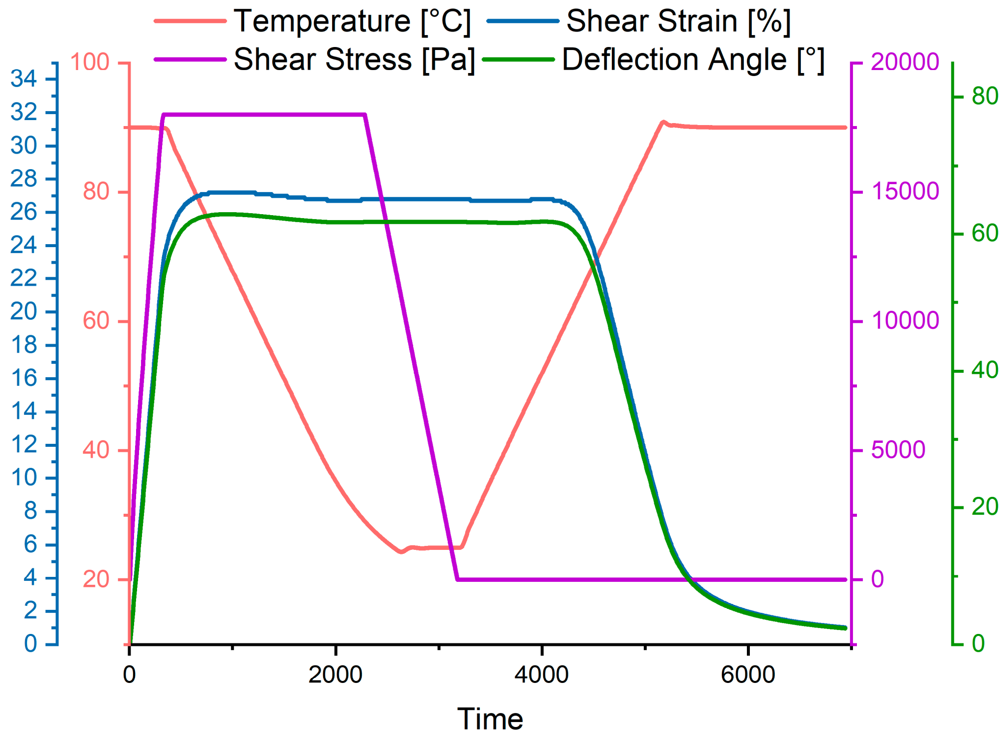

Figure S54. First cycle of the cyclo mechanic test of the metallopolymer networks **P8-Zn**.

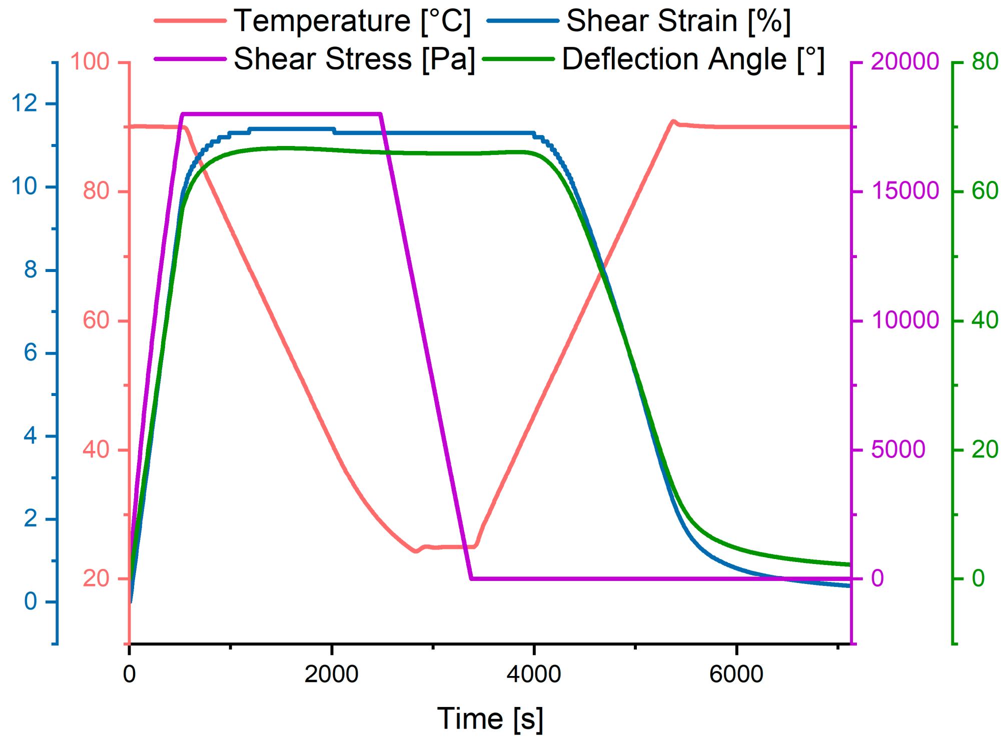

Figure S55. First cycle of the cyclo mechanic test of the metallopolymer networks **P11-Zn**.
